# Mitochondria-Targeted DNA Repair Glycosylase hOGG1 Protects Against HFD-Induced Liver Oxidative Mitochondrial DNA Damage and Insulin Resistance in OGG1-Deficient Mice

**DOI:** 10.3390/ijms252212168

**Published:** 2024-11-13

**Authors:** Larysa V. Yuzefovych, Hye Lim Noh, Sujin Suk, Anne Michele Schuler, Madhuri S. Mulekar, Viktor M. Pastukh, Jason K. Kim, Lyudmila I. Rachek

**Affiliations:** 1 Departments of Pharmacology, Frederick P. Whiddon College of Medicine, University of South Alabama, Mobile, AL 36688, USA; 2 Program in Molecular Medicine, University of Massachusetts Chan Medical School, Worcester, MA 01605, USA; 3 Departments of Microbiology, Frederick P. Whiddon College of Medicine, University of South Alabama, Mobile, AL 36688, USA; 4 Department of Mathematics and Statistics, College of Art and Science, University of South Alabama, Mobile, AL 36688, USA; 5 Division of Endocrinology, Metabolism and Diabetes, Department of Medicine, University of Massachusetts Chan Medical School, Worcester, MA 01605, USA

**Keywords:** Ogg1, insulin resistance, obesity, high-fat diet, liver, mitochondrial DNA damage, gluconeogenesis, DNA methylation

## Abstract

8-oxoguanine DNA glycosylase-1 (OGG1) is a DNA glycosylase mediating the first step in base excision repair which removes 7,8-dihydro-8-oxoguanine (8-oxoG) and repairs oxidized nuclear and mitochondrial DNA. Previous studies showed that OGG1 deficiency results in an increased susceptibility to high-fat diet (HFD)-induced obesity and metabolic dysfunction in mice, suggesting a crucial role of OGG1 in metabolism. However, the tissue-specific mechanisms of how OGG1 deficiency leads to insulin resistance is unknown. Thus, in the current study, we used a hyperinsulinemic-euglycemic clamp to evaluate in-depth glucose metabolism in male wild-type (WT) mice and *Ogg1−/−* (*Ogg1-KO*) mice fed an HFD. *Ogg1-KO* mice fed HFD were more obese, with significantly lower hepatic insulin action compared to WT/HFD mice. Targeting human OGG1 to mitochondria protected against HFD-induced obesity, insulin resistance, oxidative mitochondrial DNA damage in the liver and showed decreased expression of liver gluconeogenic genes in *Ogg1-KO* mice, suggesting a putative protective mechanism. Additionally, several subunits of oxidative phosphorylation protein levels were noticeably increased in *Ogg1-KO/Tg* compared to *Ogg1-KO* mice fed an HFD which was associated with improved insulin signaling. Our findings demonstrate the crucial role of mitochondrial hOGG1 in HFD-induced insulin resistance and propose several protective mechanisms which can further direct the development of therapeutic treatment.

## 1. Introduction

High dietary fat intake leads to obesity and thus to insulin resistance, and this represents a major risk factor for metabolic syndrome, type 2 diabetes, and cardiovascular disorders, such as coronary artery disease and heart failure. Increasing evidence accumulated over the last decade indicates that oxidative stress and mitochondrial dysfunction have been implicated in insulin resistance [1,2] but the underlying mechanisms are still unknown. Oxidative stress causes damage to proteins, lipids, and DNA. The oxidation of guanine to 7,8-dihydro-8-oxoguanine (8-oxoG) is the most common type of oxidative DNA lesion which is also considered as a marker of oxidative stress [3]. 8-oxoG DNA glycosylase-1 (OGG1) is a DNA glycosylase mediating the first step in base excision repair (BER) which removes 8-oxoG and repairs oxidized nuclear and mitochondrial DNA (mtDNA) [4,5]. OGG1 has both nuclear and mitochondrial localization [6]. Previously, our group has shown that protection of mtDNA from palmitate-induced damage by overexpression of human OGG1 (hOGG1) targeted to mitochondria significantly diminished palmitate-induced mitochondrial reactive oxygen species and mitochondrial dysfunction and, thus, restored insulin signaling in vitro [7,8]. OGG1 deficiency results in an increased susceptibility to high fat diet (HFD)-induced obesity, fatty liver, metabolic dysfunction, and glucose intolerance in mice, suggesting a crucial role of OGG1 in glucose metabolism [9]. Conversely, enhanced expression of mitochondrial hOGG1 (mt-hOGG1) makes mice resistant to obesity and adiposity by altering mitochondrial energetics in white adipose tissue [10]. Moreover, several studies have reported that the Ser326Cys gene in hOGG1 polymorphism is associated with insulin resistance [11], obesity [12], and type 2 diabetes [13,14]. Considering that lack of OGG1 contributes to obesity and metabolic dysfunction in mice, we were interested in (1) determining tissue-specific insulin resistance in *Ogg1−/−* (*Ogg1-KO*) mice; and (2) evaluating the contribution of mt-hOGG1 alone to whole-body insulin resistance in mice.

In the current study, we performed a 2 h hyperinsulinemic-euglycemic clamp to measure tissue-specific insulin sensitivity in wild-type (WT) and *Ogg1-KO* mice after chronic feeding of a low-fat diet (LFD as controls) or an HFD. The results of the clamp study showed a strong trend of increased insulin resistance with lower glucose infusion rates and whole-body glucose turnover and glycogen synthesis in HFD-fed *Ogg1-KO* mice compared to HFD-fed WT mice. Additionally, our results demonstrated that hepatic insulin action was significantly lower in the HFD-fed *Ogg1-KO* mice which is consistent with recent evidence showing Ogg1 regulation of hepatic gluconeogenesis in the fed state [15].

In the view that OGG1 is predominantly important to the maintenance of mtDNA integrity [15] and mitochondrial function and metabolism is crucial to insulin resistance, we were particularly interested to evaluate the effect of the absence of nuclear OGG1 and mitochondrial reconstitution of hOGG1 on insulin resistance. To perform this study, we utilized a novel mouse model which lacks endogenous mouse OGG1, both nuclear and mitochondrial (*Ogg1-KO*), but overexpresses exogenous mitochondrial targeted hOGG1 (hOGG1, subunit 1-α; *Ogg1-KO/Tg*) [16]. Previously, using this mouse model, Komakula et al. showed that mt-hOGG1 alone attenuates HFD-induced weight gain in *Ogg1-KO* mice [10]. Additionally, our previous study demonstrated that *Ogg1-KO/Tg/PyMT* mice were protected from breast cancer progression and metastasis [17]. Using *Ogg1-KO/Tg* mice, we showed that mitochondrial delivery of hOGG1 significantly lowers oxidative mtDNA damage and thus, obesity, hyperglycemia, and insulin resistance phenotype in HFD-fed *Ogg1-KO* mice. Our findings suggest that mt-hOGG1 but not nuclear OGG1 expression is crucial to prevent insulin resistance and provides a strong rationale to deliver hOGG1 and possibly other DNA repair enzymes into mitochondria as a new translational approach for treatment of insulin resistance.

## 2. Results

### 2.1. Insulin Sensitivity and Glucose Metabolism During Hyperinsulinemic-Euglycemic Clamp in Ogg1-KO Mice

**Figure 1:**
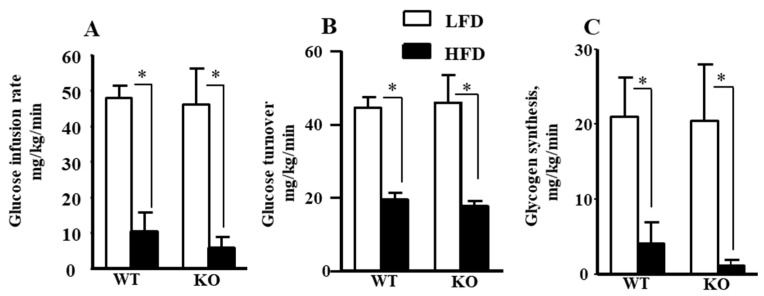
Glucose metabolism in WT and *Ogg1-KO* mice after 16 weeks of LFD or HFD feeding. (**A**) Steady-state glucose infusion rate during hyperinsulinemic-euglycemic clamp. Values are the mean ± standard deviation (SD), n = 5–8 per group. (**B**) Whole-body glucose turnover. Values are the mean ± SD, n = 5–8 per group. (**C**) Whole-body glycogen synthesis. Values are the mean ± SD, n = 5–7 per group, * *p* < 0.05. Statistical analysis can be found in Supplementary File S1.

**Figure 2:**
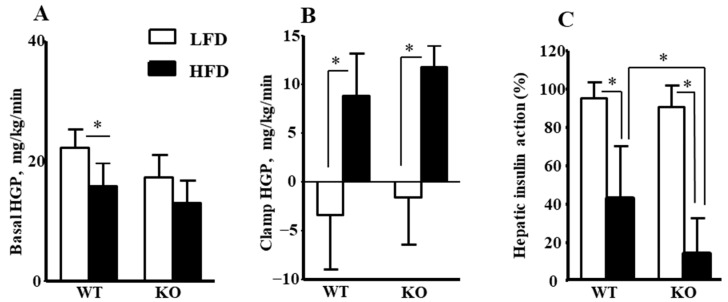
HGP and hepatic insulin action during hyperinsulinemic-euglycemic clamp in awake WT and *Ogg1-KO* mice fed LFD or HFD for 16 weeks. (**A**) Basal HGP. (**B**) Insulin-stimulated HGP during clamping. (**C**) Hepatic insulin action was calculated as insulin-mediated percent suppression of basal hepatic glucose production during clamps. Values are the mean ± SD, n = 5–8 per group, * *p* < 0.05. Statistical analysis can be found in Supplementary File S1.

To determine the metabolic organs responsible for insulin resistance in *Ogg1-KO* mice, insulin action and glucose metabolism were assessed during a 2 h hyperinsulinemic-euglycemic clamp in awake age-matched male WT and *Ogg1-KO* mice following 16 weeks of HFD or LFD. During the clamps, plasma glucose concentration was maintained at ~120 mg/dL by a variable rate of glucose infusion in all groups. As shown in Figure 1A, *Ogg1-KO/*HFD mice had a strong trend toward lower glucose infusion rates during clamps when compared to WT mice fed an HFD, suggesting increased insulin resistance in *Ogg1-KO*/HFD mice. Mean whole-body glucose turnover did not differ between groups, but mean whole-body glycogen synthesis tended to be reduced in HFD-fed *Ogg1-KO* mice compared to HFD-fed WT mice although the difference was not statistically significant (Figure 1B,C). Basal hepatic glucose production (HGP) did not differ between groups, but clamp HGP tended to be higher in *Ogg1-KO* mice on HFD (Figure 2A,B). As a result, hepatic insulin action, expressed as insulin-mediated percent suppression of basal HGP, was significantly reduced in *Ogg1-KO* mice fed with an HFD compared to WT mice fed with an HFD (Figure 2C).

### 2.2. Insulin-Stimulated Glucose Uptake in Skeletal Muscle and Adipose Tissue in Ogg1-KO Mice

Insulin-stimulated glucose uptake in skeletal muscle was not altered significantly in *Ogg1-KO* mice compared to WT mice on the respective diets (Supplementary Figure S1A), although we cannot rule out that we are missing differences in skeletal muscle insulin action that may exist between groups due to the physiological insulin dose used during the clamp. The insulin dose for clamp experiments was chosen to allow comparison to the LFD-fed mice, which may have compromised our ability to properly assess insulin resistance in the HFD-fed animals. Although not statistically significant, insulin-stimulated glucose uptake in white adipose tissue (WAT) was observed to be higher in *Ogg1-KO*/HFD mice compared to WT/HFD mice, and this is consistent with increased adiposity in *Ogg1-KO* mice after HFD (Supplementary Figure S1B). These data demonstrate that *Ogg1-KO* mice tended to be more insulin-resistant than WT mice after HFD, and this increased insulin resistance is selective to the liver.

### 2.3. Targeting of hOGG1 into Mitochondria of Ogg1-KO Mice Reduced HFD-Induced Obesity, Hyperglycemia, and Insulin Resistance Phenotype

Consistent with the previous study [9], after 16 weeks of an HFD, the *Ogg1-KO* mice at University of Massachusetts Mouse Metabolic Phenotyping Center (UMass MMPC) facility displayed a greater obese phenotype as compared to WT mice fed HFD (Supplementary Figure S2A) with significant increases in whole-body fat mass but no significant difference in lean body mass compared to WT/HFD mice (Supplementary Figure S2B,C). Both WT and *Ogg1-KO* mice fed with an LFD showed comparable mean body weight (Supplementary Figure S2A). *Ogg1-KO* mice fed LFD showed a trend toward lower lean body mass (*p* = 0.1218, Supplementary Figure S1B) and higher fat mass (*p* = 0.1231, Supplementary Figure S1C) compared to WT/LFD mice although neither difference was significant.

**Figure 3:**
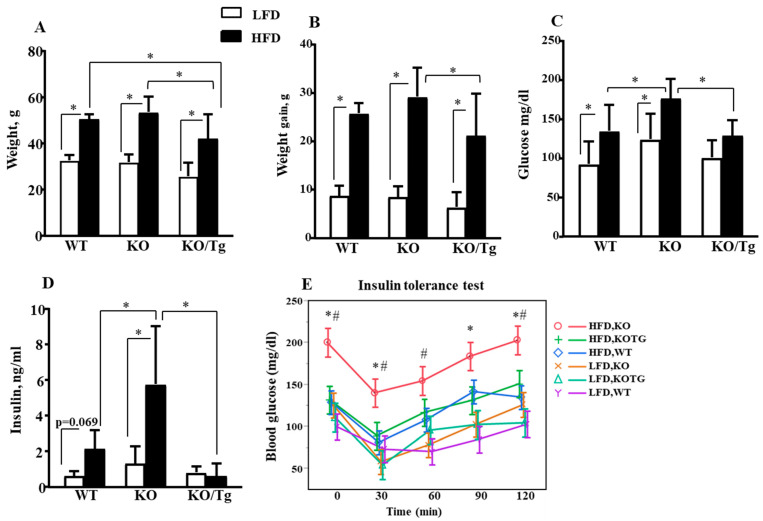
Targeting hOGG1 to mitochondria protected against HFD-induced obesity, hyperglycemia, and whole-body insulin resistance in *Ogg1-KO* mice. (**A**) Body weight, (**B**) weight gain, (**C**) fasted glucose, and (**D**) insulin levels in WT, *Ogg1-KO* mice, and *Ogg1-KO/Tg* mice after 16 weeks of LFD or HFD feeding. Values are the mean ± SD, n = 11–16 per group for (**A**–**C**) and n = 9–15 for (**D**), * *p* < 0.05. For insulin levels (**D**) there is an interaction (diet and genotype), *p* < 0.0001. (**E**) Insulin tolerance tests in WT, *Ogg1-KO*, and *Ogg1-KO/Tg* mice after 16 weeks of LFD or HFD feeding. Values are the mean ± SD, n = 11–16 per group, * *p* < 0.05 vs. respective time point in the HFD-fed *Ogg1-KO/Tg* group; # *p* < 0.05 vs. respective time point in the HFD-fed WT group. Statistical analysis can be found in Supplementary File S2.

Results of long-term HFD feeding demonstrated that targeting of hOGG1 into mitochondria of *Ogg1-KO* mice reduced HFD-induced obesity (Figure 3A,B). *Ogg1-KO* mice fed HFD at University of South Alabama’s animal facility showed a slight non-significant increase in weight and weight gain compared to WT/HFD (Figure 3A,B). HFD-fed *Ogg1-KO* mice demonstrated significantly increased fasted levels of glucose and insulin compared to both WT and *Ogg1-KO/Tg* mice fed with an HFD (Figure 3C,D). Additionally, results of ITT showed that HFD-fed *Ogg1-KO* mice were more insulin-resistant compared to WT mice fed an HFD (Figure 3E). Importantly, targeting of hOGG1 into mitochondria of *Ogg1-KO* mice reduced HFD-induced whole-body insulin resistance phenotype in *Ogg1-KO/Tg* mice as evidenced by a decrease in insulin levels as well as in results of insulin tolerance test (ITT, Figure 3D,E). Interestingly, insulin levels in HFD-fed *Ogg1-KO/Tg* mice tended to be lower compared to HFD-fed WT mice, although the difference was not statistically significant (*p* = 0.0812, Figure 3D).

### 2.4. Targeting of hOGG1 into Mitochondria of Ogg1-KO Mice Protected Against Oxidative mtDNA Damage, Altered mtDNA Content, and Expression of OXPHOS Proteins in the Liver

**Figure 4:**
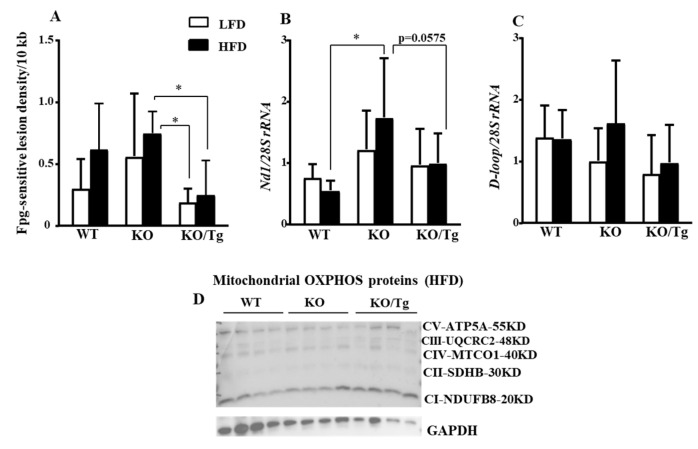
Analysis of oxidative mtDNA damage, mtDNA content, and mitochondrial oxidative phosphorylation (OXPHOS) proteins levels in WT, *Ogg1-KO*, and *Ogg1-KO/Tg* mice. (**A**) Targeting hOGG1 to mitochondria protected against HFD-induced oxidative mtDNA damage in the liver in *Ogg1-KO* mice. Liver oxidative mtDNA damage is presented as changes in oxidative lesion density calculated as a -*ln* of the quotient of hybridization intensities in bands from Fpg-treated and untreated samples normalized to 10 kb. Values are mean ± SD, n = 5–8, * *p* < 0.05. (**B**) *Nd1* and (**C**) *D-loop* mtDNA abundance in the liver. Values are mean ± SD, n = 9–12, * *p* < 0.05. Statistical analysis can be found in Supplementary File S2. (**D**) Expression level of mitochondrial OXPHOS proteins in HFD-fed WT, *Ogg1-KO*, and *Ogg1-KO/Tg* mice. GAPDH was used as loading control. A representative of two independent experiments is shown.

First, using the quantitative alkaline Southern blot and formamidopyrimidine glycosylase (Fpg)-sensitive assay as described in the Materials and Methods Section , we evaluated oxidative mtDNA in the liver. We found that targeting of hOGG1 into mitochondria of *Ogg1-KO* mice protected against oxidative mtDNA damage in the liver (Figure 4A) of *Ogg1-KO/Tg* mice, fed both LFD and HFD. Second, we evaluated mtDNA content (mtDNA/nuclear DNA (nDNA) ratio) using total DNA isolated from liver and mtDNA sequences for NADH dehydrogenase subunit 1 (ND1) and D-loop (displacement loop). As shown in Figure 4B, HFD-fed *Ogg1-KO* mice have a significant compensatory increase in mtDNA content in the coding region of *Nd1* compared to WT/HFD mice. *Nd1* content was reduced (*p* = 0.0575) in *Ogg1-KO/Tg* mice fed HFD compared to *Ogg1-KO* fed HFD. *D-loop* mtDNA content in the liver showed a slight non-significant increase in *Ogg1-KO* fed HFD but a decrease in *Ogg1-KO* fed LFD compared to WT mice (Figure 4C).

Due to the perplexing levels of mtDNA, we hypothesized that reduced oxidized mtDNA damage as well as mtDNA content may be due to improved mitophagy or mitochondrial biogenesis. Since both mitophagy and mitochondrial biogenesis can regulate mtDNA abundance and damage, we evaluated the expression of markers of mitophagy (PTEN-induced kinase 1; PINK1, Supplementary Figure S3A) and mitochondria biogenesis (peroxisome proliferator-activated receptor-gamma coactivator 1-alpha; PGC-1α, Supplementary Figure S3B). It is possible that the lower oxidative mtDNA damage in *Ogg1-KO/Tg* was likely due to excessive removal of damaged molecules by mitophagy, as the copy number of mtDNA was lower (*p* = 0.0574, Figure 4B) and *Pink1* expression was slightly higher in *Ogg1-KO/Tg*/LFD but not in *Ogg1-KO/Tg*/HFD mice (Supplementary Figure S3A). At the same time, peroxisome proliferator-activated receptor-gamma coactivator 1-alpha (PGC-1*a*) expression, the marker of mitochondrial biogenesis, was upregulated in *Ogg1-KO* fed an HFD, while mt-hOGG1 decreased *Pgc-1*α expression in *Ogg1-KO/Tg* mice and was comparable to WT/HFD mice (Supplementary Figure S3B).

Since mtDNA encodes 13 proteins subunits involved in OXPHOS, we next evaluated the expression of OXPHOS in WT, *Ogg1-KO*, and *Ogg1-KO/Tg* mice fed HFD or LFD. We found that the increased oxidative mtDNA damage and observed changes in mtDNA content were associated with alteration in the proteins of OXPHOS chain in *Ogg1-KO* mice fed HFD (Supplementary Figure S4). Consistent with an increase in mtDNA level in *Ogg1-KO* mice, HFD also increased protein levels of several subunits of the OXPHOS compared to the LFD-fed mice (Supplementary Figure S4). This increase was also observed for extracts isolated from *Ogg1-KO/Tg*/HFD but not for the ones isolated from WT/HFD mice (Supplementary Figure S4). The expression level of subunit NDUDB8 of complex I was reduced in *Ogg1-KO* mice fed LFD compared to *Ogg1-KO* fed HFD (Supplementary Figure S4). At the same time, the protein levels of several subunits of OXPHOS complexes were non-significantly increased in HFD-fed *Ogg1-KO/Tg* mice compared to *Ogg1-KO*/HFD mice (Figure 4D, quantified in Supplementary Figure S5).

### 2.5. Targeting of hOGG1 into Mitochondria of Ogg1-KO Mice Improved Insulin Signaling, Altered Expression of Gluconeogenesis Genes and Methylation in DNA

**Figure 5:**
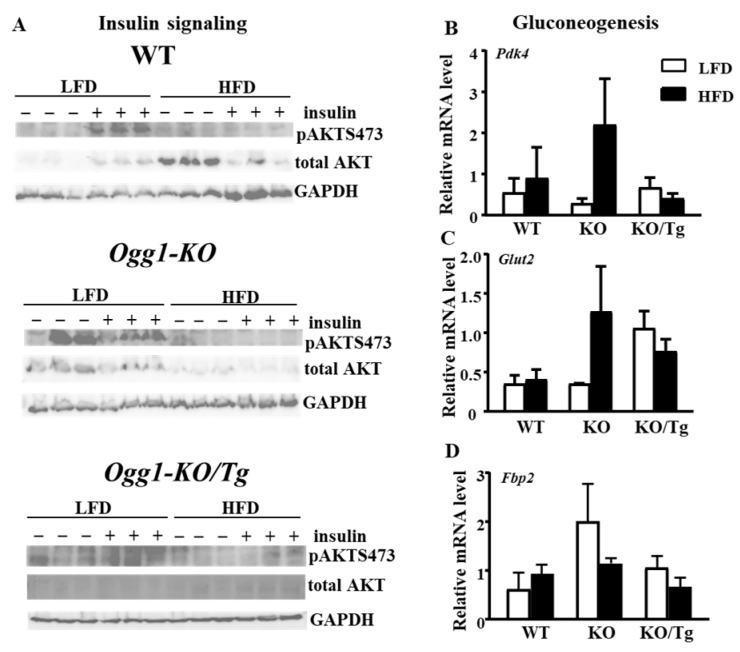
Insulin signaling and mRNA levels of gluconeogenesis genes in WT, *Ogg1-KO*, and *Ogg1-KO/Tg* mice. (**A**) Immunoblot analysis for AKT (pSer473) in the liver from LFD/HFD-fed mice after ip injection of 1.5 U/kg insulin for 5 min. GAPDH was used as loading control. Expression of the following gluconeogenesis genes (**B**) *Pdk4*, (**C**) *Glut2*, and (**D**) *Fbp2* was evaluated by quantitative reverse transcription PCR in the liver from LFD- or HFD-fed mice. Values are the mean ± standard error (SE), n = 5–6 per group. For *Glut2* expression level, there was an interaction (diet and genotype), *p* = 0.047. Statistical analysis can be found in Supplementary File S2.

Consistent with improved insulin resistance, mitochondrial hOGG1 improved insulin signaling as shown by increased insulin-stimulated phosphorylation of pAKTSer473 in HFD-fed *Ogg1-KO/Tg* compared to *Ogg1-KO*/HFD mice, although the difference was not significant (Figure 5A, quantified in Supplementary Figure S6, A, *p* = 0.4416). Interestingly, HFD significantly increased the total level of AKT in WT mice compared to LFD-fed mice (Figure 5A, quantified in Supplementary Figure S6, B). The similar trend was observed for *Ogg1-KO/Tg*/HFD mice compared to *Ogg1-KO/Tg*/LFD mice (Supplementary Figure S6, *p* = 0.082). On the contrary, the total level of AKT was non-significantly decreased in *Ogg1-KO*/HFD compared to *Ogg1-KO*/LFD mice (Supplementary Figure S6, *p* = 0.256).

Since a previous study demonstrated that OGG1 controls hepatic gluconeogenesis in mice fed a regular diet [15], we performed quantitative reverse transcription PCR analysis of total liver RNA using primers specific for several gluconeogenesis genes. Quantitative reverse transcription PCR analysis demonstrated a parallel non-significant increase in pyruvate dehydrogenase kinase 4 (PDK4), fructose-bisphosphatase 2 (FBP2), and glucose transporter 2 (GLUT2) transcripts (GLUT2: interaction, *p* = 0.0475, genotype *p* = 0.0673, Figure 5B) in *Ogg1-KO*/HFD mice compared to WT/HFD mice. Delivery of mt-hOGG1 to *Ogg1-KO* mice reduced the observed increase in all three transcripts, although the changes were not statistically significant because of the small sample size used for this experiment (Figure 5B).

8-OxoG may be an epigenetic marker linked to DNA methylation [18]. To determine if the observed changes in the phenotypes of *Ogg1-KO*, WT, and *Ogg1-KO/Tg* mice are associated with alteration in DNA methylation, we performed 5-methylcytosine (5-mC) analysis of total DNA isolated from WT, *Ogg1-KO*, and *Ogg1-KO/Tg* mice. While HFD increased epigenetic DNA methylation marks (5-mC) in both WT and *Ogg1-KO* mice, the level of 5-mC was greater in DNA from *Ogg1-KO*/HFD mice (Supplementary Figure S7). Staining patterns for 5-mC in DNA from *Ogg1-KO/Tg* were distinct from WT and *Ogg1-KO* mice, as HFD decreased 5-mC DNA methylation compared to LFD--fed mice in *Ogg1-KO/Tg* mice (Supplementary Figure S7).

## 3. Discussion

Our understanding of the role of mtDNA damage and repair in the development of obesity and metabolic syndrome has been advanced in the last several years. Simultaneously, our knowledge about OGG1, the major enzyme of BER, has been evolving, with the addition of new functions to this enzyme, beyond BER [19]. While numerous studies showed that lack of OGG1 in mice contributes to obesity and metabolic dysfunction, the precise tissue-specific mechanisms of insulin resistance in *Ogg1-KO* mice remain unknown. The novelty of our current work is that we demonstrated: (1) HFD-induced insulin resistance is primarily due to defects in hepatic insulin action in *Ogg1-KO* mice and (2) reconstitution of the hOGG1 enzyme specifically in mitochondria of *Ogg1-KO* animals not only prevented oxidative mtDNA damage but also coordinated the protection from HFD-induced insulin resistance.

Previously, it has been shown that lack of OGG1 is associated with the development of obesity and metabolic syndrome [9]. Our group has shown that protection of mtDNA against palmitate-induced damage by overexpression of hOGG1 targeted to mitochondria significantly diminished palmitate-induced oxidative stress and mitochondrial dysfunction and, thus, restored insulin signaling in vitro [7,8]. Also, a recent study showed that OGG1 plays a critical role in modulating mitochondrial energetics in WAT and whole-body energy balance, thus protecting against obesity in mt-hOGG1-overexpressing transgenic WT mice [10]. Furthermore, OGG1 plays a protective role in HFD-induced atherogenesis by preventing excessive inflammasome activation [20] and mt-hOGG1 expression alone provides a protective role in age-associated inflammation [21]. Also, clinical studies showed that Ser326Cys polymorphism in hOGG1 is associated with insulin resistance, obesity, and type 2 diabetes [11,12,13,14].

A previous study showed that *Ogg1-KO* mice fed a normal diet ad libitum exhibited hyperglycemia, elevated insulin levels, higher liver glycogen content, and inefficient suppression of gluconeogenesis [15]. Furthermore, *Ogg1-KO* mice exhibited reduced mitochondrial electron transport chain capacity and a combined low activity of the pyruvate dehydrogenase complex, pointing to inefficient channeling of glycolytic end-products into the citric acid cycle [15]. These data demonstrate a physiological role of BER that goes beyond DNA maintenance, and further implies that DNA repair is involved in regulating metabolism. Despite all these previous studies, there were, however, many gaps in knowledge regarding the tissue-specific mechanisms of how OGG1 deficiency leads to whole-body insulin resistance. Our current study showed that hepatic insulin action was significantly lower in the HFD-fed *Ogg1-KO* mice (Figure 2C) which was consistent with recent evidence showing OGG1 regulation of hepatic gluconeogenesis in the fed state [15]. This is the first evidence demonstrating that OGG1 contributes to HFD-induced insulin resistance in the liver. As HFD-fed *Ogg1-KO* mice showed a big increase in fat mass, we do not know whether OGG1 expression in the liver directly influenced insulin resistance or whether these differences were secondary, possibly due to the differences in the body weight between the WT and *Ogg1-KO* mice on HFD.

The second novelty of our current work is that nuclear OGG1 is nonessential for insulin resistance phenotype, since specific reconstitution of mt-hOGG1 alone prevented HFD-induced insulin resistance in OGG1-deficient animals. Indeed, mt-hOGG1 expression significantly attenuated oxidative mtDNA damage in the liver (Figure 4A) and protected from obesity, hyperglycemia, and insulin resistance phenotype in HFD-fed *Ogg1-KO* mice (Figure 3A–E). Furthermore, consistent with the study by Komakula et al. [10], HFD-fed *Ogg1-KO/Tg* mice were significantly less obese compared to both *Ogg1-KO* and WT mice fed an HFD (Figure 3A). As we mentioned above, while mt-hOGG1 expression significantly decreased the weight of HFD-fed *Ogg1-KO* mice (Figure 3A,B), there was an inter-laboratory discrepancy in the mouse’s weight in *Ogg1-KO*/HFD compared to WT/HFD mice (Supplementary Figure S2A). *Ogg1-KO* mice maintained at the UMass animal facility gained significant weight compared to WT mice fed an HFD (46.857 + 2.34 vs. 41.625 + 4.173, *p* = 0.025, Supplementary Figure S2A), whereas the *Ogg1-KO*/HFD maintained at the animal facility at University of South Alabama were also heavier than WT/HFD but the difference in weight was not significant (53.49 + 6.74 vs. 50.47 + 2.17, *p* = 0.7445, Figure 3A). Although the diet and its duration was the same for both groups and both groups of mice have the same C57Bl6J background, the difference in weight gain can be explained by the inter-laboratory specifics in mouse handling. As it has been mentioned in the recent review [19], previous concerns about inter-laboratory discrepancies in OGG1 mouse phenotypes were reported, which can be explained by inter-institutional difference in the intestinal microbiome and its influence on organismal health.

Consistent with the increased hepatic insulin resistance, HFD-fed *Ogg1-KO* mice showed increased whole-body insulin resistance phenotype as evidenced by an increase in insulin levels as well as in the results of ITT (Figure 3D,E). Outstandingly, mt-hOGG1 expression alone was sufficient enough to prevent both hyperglycemia and insulin resistance in *Ogg1-KO* mice (Figure 3C–E). The exact mechanisms by which mt-hOGG1 protects against HFD-induced insulin resistance are unclear, however, based on the results of oxidative mtDNA damage analysis, we can propose that the OGG1 BER enzyme activity is involved since mt-hOGG1 prevented liver oxidative mtDNA damage in *Ogg1-KO/Tg* mice (Figure 4A). We can only speculate that mt-hOGG1 activates mitophagy, since it is possible that lower oxidative mtDNA damage in *Ogg1-KO*/Tg mice was likely due to excessive mitophagic removal of damaged molecules, as the copy number of mtDNA was lower (*p* = 0.0574, Figure 4B) and *Pink1* expression was slightly higher in *Ogg1-KO/Tg*/LFD but not in *Ogg1-KO/Tg*/HFD mice (Supplementary Figure S3A). At the same time, *Pgc*-1α expression was lower in HFD-fed *Ogg1-KO/Tg* mice and comparable to WT/HFD mice, probably indicating reduced biogenesis compared to HFD-fed *Ogg1-KO* mice (Supplementary Figure S3B). In contrast to the previous findings by Sampath et al. [9], we found that HFD-fed *Ogg1-KO* have significantly increased mtDNA content compared to HFD-fed WT mice (*Nd1* content, Figure 4B). Interestingly, *Nd1* content tended to be reduced in HFD-fed *Ogg1-KO/Tg* mice compared to *Ogg1-KO* fed HFD. On the other hand, *D-loop* mtDNA content in the liver showed a slight non-significant increase in HFD-fed *Ogg1-KO* but a decrease in LFD-fed *Ogg1-KO* compared to WT mice (Figure 4C). Mitochondrial expression of hOGG1 tended to decrease mtDNA content compared to *Ogg1-KO* mice (Figure 4B,C). We speculate that the differences in mtDNA abundance in the liver between *Ogg1-KO* mice in our and previous study [9] can be influenced by the percent of C57Bl/6J background, since mice in the study [9] were of pure C57Bl/6J background, as opposed to the mixed C57Bl6J/FVBNJ background of mice used in our study. 

Our current study demonstrated that protein levels of several subunits of OXPHOS complexes tended to be increased in HFD-fed *Ogg1-KO/Tg* mice compared to HFD-fed *Ogg1-KO* mice (Figure 4D, quantified in Supplementary Figure S5). Mechanistically, it may propose one of the mechanisms of protection against insulin resistance, especially since it was shown that NDUFAB1 protects against HFD-induced obesity and insulin resistance by enhancing mitochondrial metabolism [22]. Similarly, a study by Komakula et al. also demonstrated that overexpression of mt-hOGG1 in WT mice has a tissue-specific effect and increased expression levels of OXPHOS proteins but only in adipose tissue which were reduced by an HFD in WT mice [10]. Taking these observations together and considering that mitochondrial metabolism function plays an important role in insulin signaling and insulin resistance, we speculate that mt-hOGG1 promotes mitochondrial well-being and quality, thus leading to the observed improved insulin signaling in the liver of HFD-fed *Ogg1-KO/Tg* mice (Figure 5A, quantified in Supplementary Figure S6A).

Suppression of gluconeogenesis can be an additional mechanism by which mt-hOGG1 protects against HFD-induced insulin resistance. Similar to the previous report [15], HFD-fed *Ogg1-KO* mice may have an inefficient suppression of gluconeogenesis as shown by a tendency to increased expression level of several gluconeogenesis genes, *Glut2*, *Pdk*4, *Fbp2* (Figure 5B), and *Pgc-1*α) (Supplementary Figure S3B) indicating stimulated gluconeogenesis in the liver of HFD-fed *Ogg1-KO*. Conversely, targeting mt-hOGG1 tended to reduce mRNA levels of *Glut2*, *Fbp2, Pdk4*, and *Pgc-1α* in the liver of HFD-fed *Ogg1-KO/Tg* mice, suggesting a putative protective mechanism by inhibiting gluconeogenesis. Curiously, for *Pdk4* and *Fbp2* genes, the expression levels in *Ogg1-KO/Tg*/HFD mice were lower than in WT/HFD mice. Although we showed an increase in *Pdk4* levels only on HFD but not on a control diet, our data are in line with a previous study which showed that overexpression of mt-hOGG1 ameliorated increased level of *Pdk4* in *Ogg1-KO* mice fed a regular rodent diet [15]. How mt-hOGG1 suppressed expression of selected gluconeogenesis genes requires further investigation.

Deoxycytidine next to 8-oxoG in CpG sequences (sites of epigenetic methylation) may be more methylated in *Ogg1-KO* mice since OGG1-dependant repair of 8-oxoG in CpG may initiate erasure of the methylation marks as has been shown previously in vitro [23]. An additional study demonstrated that OGG1 is crucial for oxidative stress-induced DNA demethylation [24]. A recent study on brain development showed that *Ogg1-KO* mice at 2–3 months of age demonstrate enhanced gene- and sex-dependent DNA damage (strand breaks) and decreased epigenetic DNA methylation marks (5-mC, 5-hydroxymethylcytosine (5-hmC)) [25]. That study suggested that OGG1 may affect the developing brain by non-mutational, epigenetic mechanisms as well as via DNA repair, dependent in part upon the Ogg1 genotype, sex, and brain region [25]. Our results showed that DNA from HFD-fed *Ogg1-KO* mice have an increased level of 5-mC compared to both HFD-fed WT and KO/Tg mice. Therefore, it is reasonable to suggest that not only nuclear OGG1 but mt-hOGG1 also prevented methylation in CpG sequences. Protective mechanisms by DNA demethylation coupled with oxidative damage repair by nuclear OGG1 are extensively discussed, explaining an additional role for 8-oxoG as an epigenetic marker and stimuli-driven roles of OGG1 in gene expression modulation [18]. On the other hand, the mechanisms of the protective effect of mt-hOGG1 on methylation of DNA are not clear and remain to be evaluated.

A recent review summarized the exceptional complexity of the potential roles of OGG1 through both its non-catalytic, damage-specific promoter binding and subsequent transcriptional modulation and its catalytic role in altering DNA structure and repair of pre-mutagenic and apoptotic-inducing base damage [26]. As highlighted in that review [26], the mechanisms of regulation of metabolic genes by OGG1 deficiency are not yet known. A study by Xian et al. demonstrated that oxidized mtDNA fragments initiated cytosolic NLRP3 inflammasome activation and also activated cGAS-STING signaling and gave rise to pro-inflammatory extracellular DNA [27]. Furthermore, both that [27] and a more recent study [21] showed that mt-hOGG1 expression decreased cytosolic mtDNA levels and prevented cGAS-STING-mediated inflammation. Certainly, additional studies are needed to determine if the observed changes in the phenotypes of *Ogg1-KO* and *Ogg1-KO/Tg* mice are associated with cytosolic or extracellular mtDNA-regulated downstream inflammatory response. 

One of the limitations of our study was the small size group in some analyses, which most likely affected the variability in the group and made these analyses underpowered. Clearly, future studies with a greater sample size are necessary to summarize these results. While our current work pointed out some putative explanatory mechanisms for protection against insulin resistance by mt-hOGG1 expression, further studies are needed to elucidate the precise mechanisms underlying the reported effects of mt-hOGG1 which potentially will have great therapeutic benefits.

In conclusion, our findings uncovered a critical role of OGG1 deficiency in promoting HFD-induced hepatic insulin resistance which led to whole-body insulin resistance in a diet-induced obesity mouse model. Given the primary role of increased hepatic gluconeogenesis in the pathogenesis of hyperglycemia in insulin resistance and type 2 diabetes, new drugs aimed at targeting OGG1 specifically to mitochondria may propose a novel form of treatment for metabolic disease.

## 4. Materials and Methods

### 4.1. Animals and Diets

This work contains two animal studies: (1) analysis of body composition and hyperinsulinemic-euglycemic clamp to assess insulin sensitivity (WT and *Ogg1-KO*), which was completed at NIH MMPC, University of Massachusetts, and (2) metabolic analysis, evaluation of oxidative mtDNA damage, protein, mRNA, and DNA methylation analysis (WT, *Ogg1-KO*, and *Ogg1-KO/Tg* mice); mice were maintained at the University of South Alabama Frederick P. Whiddon COM, Department of Comparative Medicine animal facility. We used mice of three genotypes with altered expression of OGG1: WT mice, *Ogg1-KO*, which lack endogenous mouse OGG1, both nuclear and mitochondrial [28]), and *Ogg1-KO* mice transgenically overexpressing hOGG1 in mitochondria (*Ogg1-KO/Tg*, also referred to as a mitochondrial “knockin” mice [16]). WT (98.61% of C57Bl/6J background), *Ogg1-KO* (97.57% of C57Bl/6J background), and *Ogg1-KO/Tg* mice (97.9% of C57Bl/6J background) of mixed C57Bl6J/FVBNJ background were backcrossed to C57Bl/6J mice (Jackson Lab (Jackson Laboratory, Bar Harbor, ME, USA)) at least 6 and 5 times, respectively. The percentage of backcrossing between the donor (FVB/NJ) and recipient (C57BL/6J) strains was provided by Jackson Lab (Jackson Laboratory, Bar Harbor, ME, USA). Male mice (8 weeks old) of both genotypes were fed with an HFD (60% fat by calories; D12492, Research Diets, New Brunswick, NJ, USA) or a control LFD (10% fat by calories; D12450J, Research Diets, New Brunswick, NJ, USA) for 16 weeks.

### 4.2. Measurement of Body Composition

The effects of diets on body composition were monitored by noninvasively measuring whole-body fat mass and lean mass using ^1^H-MRS (Echo Medical Systems, Houston, TX, USA) at UMass MMPC as previously described [29].

### 4.3. Hyperinsulinemic-Euglycemic Clamp to Assess Insulin Sensitivity

Following LFD or HFD for 16 weeks, a survival surgery was performed at 5–6 days before clamp experiments to establish an indwelling catheter in the jugular vein. On the day of the clamp experiment, mice were fasted overnight (~15 h), and a 2 h hyperinsulinemic-euglycemic clamp was conducted in awake mice with a primed and continuous infusion of human insulin (150 mU/kg body weight priming followed by 2.5 mU/kg/min; Novolin, Novo Nordisk, Plainsboro, NJ, USA). To maintain euglycemia, 20% glucose was infused at variable rates during clamps. Whole-body glucose turnover was assessed with a continuous infusion of [3-^3^H]glucose (PerkinElmer, Waltham, MA, USA), and 2-deoxy-D-[1-^14^C]glucose (2-[^14^C]DG) was administered as a bolus (10 μCi) at 75 min after the start of clamps to measure insulin-stimulated glucose uptake in skeletal muscle (gastrocnemius) and WAT (epididymal). At the end of the clamps, mice were anesthetized, and tissues were taken for biochemical analysis as previously described [29].

### 4.4. Biochemical Analysis of Glucose Metabolism

Glucose concentrations during clamps were analyzed using 10 μL plasma by a glucose oxidase method using an Analox GM9 Analyser (Analox Instruments Ltd., Hammersmith, London, UK). Plasma concentrations of [3-^3^H]glucose, 2-[^14^C]deoxyglucose (DG), and ^3^H_2_O were determined following deproteinization of plasma samples as previously described [29]. Whole-body glucose turnover, glycolysis, and hepatic insulin action were calculated as previously described [29]. For the determination of tissue 2-[^14^C]DG-6-phosphate (2-[^14^C]DG-6-P) content, tissue samples were homogenized, and the supernatants were subjected to an ion exchange column to separate 2-[^14^C]DG-6-P from 2-[^14^C]DG. Insulin-stimulated glucose uptake in individual tissues was assessed by determining the tissue content of 2-[^14^C]DG-6-P and plasma 2-[^14^C]DG profile.

### 4.5. Measurement of Metabolic Parameters and Insulin Signaling Experiments

Fasted blood glucose was measured using a FreeStyle Lite Glucometer (Abbott Diabetes Care Inc., Alameda, CA, USA) after 4 h of food deprivation. For the ITT after 4 h of food deprivation, 0.5 u/kg of insulin (Humulin R U-100; Lilly USA, Indianapolis, IN, USA) was injected intraperitoneally (ip) into mice. Blood samples were taken from the tail at indicated times and blood glucose concentrations were measured using a Free Style Lite Glucometer (Abbott Diabetes Care Inc., Alameda, CA, USA). Blood was sampled from the tail vein in un-anesthetized mice at 0, 15, 30, 90, and 120 min after insulin administration. Blood glucose concentrations were measured using a FreeStyle Lite Glucometer (Abbott Diabetes Care Inc., Alameda, CA, USA) and animals were treated with oral or ip glucose and removed from study if the blood glucose dropped below 40 mg/dL. Serum was isolated using Microtainer Serum Separating Tubes (BD, Franklin Lakes, NJ, USA) according to the manufacturer’s instructions and frozen immediately and stored at −80 °C until analyzed. Fasted insulin concentrations were determined using an Ultra Sensitive Mouse Insulin Elisa Kit (Crystal Chem Inc., Elk Grove Village, IL, USA). For insulin signaling experiments, at the end of the 16 weeks, mice were fasted for 4–5 h prior to experiments, anesthetized with sodium pentobarbital (50 mg/kg) before an ip injection of insulin (1.5 u/kg of Humulin R U-100; Lilly USA, Indianapolis, IN, USA) or saline (basal measures). Five minutes after injection, liver tissues were excised, flash frozen in liquid nitrogen, and stored in −80 °C until processing.

### 4.6. Analysis of Oxidative mtDNA Damage and mtDNA Abundance

Oxidative mtDNA damage in the liver was evaluated using quantitative alkaline Southern blot analysis, as previously described [30,31] with some modifications to detect Fpg-sensitive oxidative base damage. Briefly, the liver was homogenized in liquid nitrogen and total DNA was isolated using a DNeasy Blood & Tissue kit (Qiagen, Valencia, CA, USA). DNA was digested with BstxI (New England Biolab, Beverly, MA, USA) and precisely quantified. To reveal oxidative base modifications, DNA was treated with Fpg (New England Biolaboratories, Beverly, MA, USA), a bacterial DNA repair enzyme that cleaves DNA at sites of oxidized purines thereby creating single-strand breaks detectable by alkaline Southern blot as described previously [30,31]. Subsequently, samples were incubated with 0.1 N NaOH for 15 min at 37 °C, mixed with loading dye, and resolved in 0.6% agarose alkaline gel. After electrophoresis, DNA was vacuum transferred to a nylon membrane and hybridized with a PCR-generated probe to the *Nd6* region of mouse mtDNA using a previously described set of primers [30] and a DIG-labeling kit (Roche Diagnostics, Mannheim, Germany). Hybridization bands images were processed with the Image J, version 1.50i software (National Institutes of Health, Bethesda, MD, USA). Changes in the equilibrium lesion density of Fpg-detectable base oxidation lesions within each experimental group were calculated as negative *ln* of the quotient of hybridization intensities in Fpg-treated and non-Fpg bands, normalized to 10 kb, and are independent of the total amount of mtDNA. The mtDNA/nDNA ratio was assessed by the quantitative real-time PCR (qRT-PCR) method using primers specific for mitochondrial *Nd1* or *D-loop* sequence (Supplementary Table S1), and normalizing for the amount of nDNA used in each reaction by using primers specific for 28S rRNA (Supplementary Table S1), which allowed a direct comparison between the amount of mtDNA and nDNA present in each sample. qRT-PCR was carried out using an iCycler iQ5 (Bio-Rad, Hercules, CA, USA) by using 20 ng of total DNA and Luna^®^ Universal qPCR Master Mix (#M3003L, NEB, Ipswich, MA, USA) according to the manufacturer’s protocol. The ratio of mtDNA to nDNA indicates relative mtDNA content per cell in the tissue.

### 4.7. Protein Isolation and Western Blot Analysis

For isolation of protein extracts from total cellular fractions, frozen liver samples were homogenized in liquid nitrogen and incubated in RIPA buffer (Fisher Scientific, Pittsburgh, PA, USA) supplemented with 0.1 mg PMSF and a 1/100 dilution of protease and phosphatase inhibitor cocktails (Sigma, St. Louis, MO, USA). Samples were centrifuged for 10 min at 14,000× *g* and the supernatants were used for Western blots as we described previously [32]. The antibodies used were rodent OXPHOS cocktail (Cat# ab110413; Abcam, Cambridge, MA, USA), pAKT, AKT, and GAPDH (Cell Signaling, Beverly, MA, USA). Complexes formed were detected with horseradish peroxidase-conjugated anti-mouse IgG or anti-rabbit IgG antibodies (Promega, Madison, WI, USA) using chemiluminescent reagents (SuperSignal, Pierce, Rockford, IL, USA) using Molecular Imager ChemiDoc XRS with Image lab software, version 3.0 (Bio-Rad Laboratories, Hercules, CA, USA). Bands images were quantified with Image J, version 1.50i software (National Institutes of Health, Bethesda, MD, USA) and intensities were normalized to GAPDH.

### 4.8. Quantitative Reverse Transcription PCR

The RNA was isolated from liver tissue using a RNeasy Plus Universal Mini kit (Qiagen) according to the manufacturer’s protocol.

Quantitative reverse transcription PCR was performed on the iCycler iQ5 (Bio-Rad, Hercules, CA, USA) by using equal amounts of total RNA according to the manufacturer’s protocol (iScript One-Step RTPCR Kit with SYBR green; Bio-Rad Laboratories, Hercules, CA, USA). Duplicate runs of each sample were normalized to abundance *28S rRNA* to determine relative expression levels. PCR primers were designed with Beacon Designer 8.2 software (Premier Biosoft International, Palo Alto, CA, USA). A complete list of primers and sequences is in Supplementary Table S2.

### 4.9. 5-Methylcytosine Levels in DNA

Levels of 5-mC in total DNA were measured as described [33] with minor modifications. Liver DNA was isolated as described above using DNeasy Blood & Tissue kit (Qiagen, Valencia, CA, USA) and treated for 1 h with RNase (DNase free; Roche Diagnostics, Mannheim, Germany) to remove any residual amount of RNA prior to DNA precipitation with ethanol. DNA samples were diluted with 2 N NaOH and 10 mM Tris-HCl, pH 8.5, and then loaded on nitrocellulose membranes (0.2 µm; Bio-Rad Laboratories, Hercules, CA, USA) using a 96-well dot-blot apparatus (Bio-Rad Laboratories, Hercules, CA, USA). After being dried at room temperature for 1 h and DNA was crosslinked to the membrane using a Spectroline XLE-1000 UV Crosslinker (Fisher Scientific, Pittsburgh, PA, USA), then blocked with 5% nonfat milk in TBST for 1 h at room temperature. Membranes were incubated in primary antibodies against 5-mC (clone 33D3; Active Motif, Carlsbad, CA, USA) at 4 °C overnight. 5-mC was visualized by using an ECL. To ensure equal loading, membranes were stained with methylene blue (0.02%) after immunoblotting.

### 4.10. Statistical Analysis

Statistical software JMP v 14.2.0 (a product of SAS Inc., Cary, NC, USA) was used for statistical analysis. Outcomes of all numerical factors were summarized using mean and SD and those for average outcomes were summarized using mean and SE, as indicated. A two-factor model with interaction was used to study the effect of diet and genotype on different outcomes and was analyzed using two-factor ANOVA with interaction. One-way ANOVA was used to compare mean outcomes for densitometry analysis of mitochondrial OXPHOS proteins. A three-factor factorial analysis (2X2X3) was conducted for densitometry analysis of Western blots for insulin signaling data. Tukey’s HSD was used for post hoc analysis. Statistical significance was defined by *p* < 0.05.

## Data Availability

Data are contained within the article or Supplementary Materials.

## Supplementary Materials

The following supporting information can be downloaded at: https://www.mdpi.com/article/10.3390/ijms252212168/s1.

## Abbreviations

BER—base excision repair; DG—2-[^14^C]deoxyglucose; 8-oxoG—7,8-dihydro-8-oxoguanine; Ogg1—8-oxoG DNA glycosylase-1/AP lyase; Fpg—formamidopyrimidine glycosylase; GLUT2—glucose transporter 2; FBP2—fructose-bisphosphatase 2; HPG—hepatic glucose production; 5-hmC—5-hydroxymethylcytosine; hOGG1—human OGG1; HFD—high-fat diet; LFD—low fat diet; ip—intraperitoneally; ITT—insulin tolerance test; 5-mC—5-methylcytosine; mt-hOGG1—mitochondrial hOGG1; mtDNA—mitochondrial DNA; ND1—NADH dehydrogenase subunit 1; nDNA—nuclear DNA; *Ogg1-KO*—Ogg1 knockout; *Ogg1-KO/Tg*—Ogg1 knockout overexpressing mitochondrial hOGG1; PGC-1α—peroxisome proliferator-activated receptor-gamma coactivator 1-alpha; PINK1—PTEN-induced kinase 1; PDK4—pyruvate dehydrogenase kinase 4; OXPHOS—oxidative phosphorylation; qRT-PCR—quantitative real-time PCR; SE —standard error; SD—standard deviation; WT—wild-type; WAT—white adipose tissue.
