# Supplementary material for: Mitochondria-Targeted DNA Repair Glycosylase hOGG1 Protects Against HFD-Induced Liver Oxidative Mitochondrial DNA Damage and Insulin Resistance in OGG1-Deficient Mice
Source: Int J Mol Sci. 2024 Nov 13;25(22):12168. doi: 10.3390/ijms252212168 (PMC11595121; doi:10.3390/ijms252212168)
Supplement: Supplementary file 1 [file ijms-25-12168-s001.zip › ijms-3233700-Proof_Suppll File1.pdf]

<u>Supplemental file #1</u>

Statistical analysis for data of body composition and hyperinsulinemic-euglycemic clamp to assess insulin sensitivity (WT and Ogg1-KO mice), which has been completed at NIH MMPC, University of Massachusetts (study #1).

Table 1. Summary statistics for outcomes and measured reported as mean difference ± SD of difference and p-value, where mean difference = mean of Group 1 – mean of Group 2.

**Group 1**	***Ogg1-KO*/LFD**	***Ogg1-KO*/LFD**	**WT/LFD**	**WT/LFD**	***Ogg1-KO*/LFD**	***Ogg1-KO*/HFD**
**Group 2**	**WT/HFD**	***Ogg1-KO*/HFD**	**WT/HFD**	***Ogg1-KO*/HFD**	**WT/LFD**	**WT/HFD**
Body weight (g)	-14.91<br>±1.70<br><0.0001*	-20.14<br>± 1.75<br>< 0.0001*	-14.83<br>±1.87<br><0.0001*	-20.06<br>±1.92<br><0.0001*	-0.09<br>±1.92<br>1.00	5.23<br>±1.70<br>0.0250*
Glucose infusion rate (mg/kg/m)	35.84<br>±3.29<br><0.0001*	40.47<br>±3.40<br><0.00001*	37.61<br>±3.62<br><0.0001*	42.24<br>±3.73<br><0.0001*	-1.77<br>±3.72<br>0.9640	-4.63<br>±3.29<br>0.5074
Hepatic Insulin Action (%)	48.13<br>±10.06<br>0.0004*	76.96<br>±10.39<br><0.0001*	52.78<br>±11.08<br>0.0005*	81.61<br>±11.38<br><0.0001*	-4.65<br>±11.38<br>0.9765	-28.82<br>±10.06<br>0.0406*
Whole body glucose turnover (mg/kg/m)	26.82<br>±2.20<br><0.0001*	28.47<br>±2.27<br><0.0001*	25.40<br>±2.42<br><0.0001*	27.05<br>±2.49<br><0.0001*	1.42<br>±2.49<br>0.9396	-1.65<br>±2.20<br>0.8751
Whole body Glycogen synthesis (mg/kg/m)	16.37<br>±2.54<br><0.0001*	19.39<br>±2.87<br><0.0001*	16.90<br>±2.80<br><0.0001*	19.92<br>±3.10<br><0.0001*	-0.53<br>±2.87<br>0.9977	-3.02<br>±2.80<br>0.7047
Fat Mass (g)	-13.29<br>±1.37<br><0.0001*	-17.78<br>±1.42<br><0.0001*	-16.88<br>±1.51<br><0.0001*	-21.37<br>±1.55<br><0.0001*	3.60<br>±1.55<br>0.1231	4.49<br>±1.37<br>0.0164*
Lean Mass (g)	-4.44<br>±0.99<br>0.0009*	-3.69<br>±1.02<br>0.0076*	-1.84<br>±1.09<br>0.3529	-1.08<br>±1.12<br>0.7694	-2.60<br>±1.12<br>0.1218	-0.76<br>±0.99<br>0.8694
Skeletal Muscle Glucose uptake	135.57<br>±58.40<br>0.1250	66.00<br>±58.40<br>0.6755	65.11<br>±68.47<br>0.7781	-4.463<br>±68.47<br>0.9999	70.46<br>±68.47<br>0.7346	69.57<br>±58.40<br>0.6388
White adipose tissue	0.39<br>±3.35	-5.88<br>±3.48	2.09<br>±3.78	-4.18<br>±3.89	-1.70<br>±3.89	6.28<br>±3.35

glucose uptake	0.9994	0.3552	0.9443	0.7080	0.9713	0.2725
