# Supplementary material for: Mitochondria-Targeted DNA Repair Glycosylase hOGG1 Protects Against HFD-Induced Liver Oxidative Mitochondrial DNA Damage and Insulin Resistance in OGG1-Deficient Mice
Source: Int J Mol Sci. 2024 Nov 13;25(22):12168. doi: 10.3390/ijms252212168 (PMC11595121; doi:10.3390/ijms252212168)
Supplement: Supplementary file 1 [file ijms-25-12168-s001.zip › ijms-3233700-Proof_Suppll file2.pdf]

Supplemental file #2

Statistical analysis for data of metabolic phenotype, evaluation of oxidative mtDNA damage, mtDNA abundance by qRT-PCR, and Quantitative Reverse Transcription PCR analysis of mRNA, which has been completed at University of South Alabama (study #2).

Statistical Methods:

All data were analyzed using JMP Pro V 16.2 (a product of SAS, Cary, NC). All numerical data was summarized using mean ± Standard deviation. Two-factor full-factorial model was used to analyze data. Post hoc analysis was conducted using Tukey's LSD test. Results were considered statistically significant for $p < 0.05$.

Results for metabolic phenotype data: Figure 3(A, B, C&D)

**Parameter**	**Factor**	**p**
**Weight, g**		
	Diet * Genotype	0.2484
	Genotype	<0.0001
	Diet	<0.0001
**Weight gain, g**		
	Diet * Genotype	0.0787
	Genotype	0.0008
	Diet	<0.0001
**Fasted glucose, mg/dl**		
	Diet * Genotype	0.3193
	Genotype	<0.0001
	Diet	<0.0001
**Insulin, ng/ml**		
	Diet * Genotype	<0.0001
	Genotype	<0.0001
	Diet	<0.0001

Results for ITT test: Figure 3E

**ITT Test**	**Factor**	**p**
Outcome		
	Time	<0.0001
	LFD/HFD	<0.0001
	WT/KO/KOTg	<0.0001
	LFD/HFD * WT/KO/KOTg	<0.0001

Results for oxidative mtDNA damage and mtDNA abundance Figure 4 (A, B, C)

**Parameter**	**Factor**	**p**
**Oxidative mtDNA damage**		
Fpg-sensitive lesion density/10 kb		
	Diet * Genotype	0.5683
	Genotype	0.0023
	Diet	0.0631
Nd1/28SrRNA		
	Diet * Genotype	0.1004
	Genotype	<0.0001
	Diet	0.4212
Dloop/28SrRNA		
	Diet * Genotype	0.2609
	Genotype	0.0399
	Diet	0.1155

Results for Quantitative Reverse Transcription PCR data for gluconeogenesis genes (Figure 5B, C&D), and *Pink1* and *Pgc-1α* (Supplemental Fig.3A&B)

**Relative mRNA level, Quantitative Reverse Transcription PCR**	**Factor**	**p**
Pdk4/28SrRNA		
	Diet * Genotype	0.1270
	Genotype	0.4048
	Diet	0.1366
Glut2/28SrRNA		
	Diet * Genotype	0.0469
	Genotype	0.0666
	Diet	0.2553
Fbp2/28SrRNA		
	Diet * Genotype	0.3213
	Genotype	0.0928
	Diet	0.3426
Pink1/28SrRNA		
	Diet * Genotype	0.5087
	Genotype	0.0246

	Diet	0.7531
Pgc-1α/28SrRNA		
	Diet * Genotype	0.5830
	Genotype	0.5348
	Diet	0.6161

Response: Weight, g

Source	LogWorth	PValue
Diet	22.219	0.00000
Genotype	6.136	0.00000
Diet*Genotype	0.605	0.24837

Summary of Fit

RSquare	0.771922
RSquare Adj	0.756084
Root Mean Square Error	5.631594
Mean of Response	39.57949
Observations (or Sum Wgts)	78

Level	- Level	Difference	Std Err Dif	Lower CL	Upper CL	p-Value
HFD,KO	LFD,KOTG	27.79091	2.401320	20.7602	34.82165	<.0001*
HFD,WT	LFD,KOTG	24.76875	2.205753	18.3106	31.22690	<.0001*
HFD,KO	LFD,KO	21.61758	2.235507	15.0723	28.16284	<.0001*
HFD,KO	LFD,WT	20.79091	2.307113	14.0360	27.54583	<.0001*
HFD,WT	LFD,KO	18.59542	2.023982	12.6695	24.52137	<.0001*
HFD,WT	LFD,WT	17.76875	2.102803	11.6120	23.92548	<.0001*
HFD,KOTG	LFD,KOTG	16.41667	2.350760	9.5340	23.29938	<.0001*
HFD,KO	HFD,KOTG	11.37424	2.350760	4.4915	18.25695	0.0001*
HFD,KOTG	LFD,KO	10.24333	2.181107	3.8573	16.62932	0.0002*
HFD,KOTG	LFD,WT	9.41667	2.254442	2.8160	16.01737	0.0011*
HFD,WT	HFD,KOTG	8.35208	2.150601	2.0554	14.64875	0.0030*
LFD,WT	LFD,KOTG	7.00000	2.307113	0.2451	13.75492	0.0379*
LFD,KO	LFD,KOTG	6.17333	2.235507	-0.3719	12.71860	0.0757
HFD,KO	HFD,WT	3.02216	2.205753	-3.4360	9.48031	0.7445
LFD,WT	LFD,KO	0.82667	2.133993	-5.4214	7.07471	0.9988

Response: Weight gain, g

Source	LogWorth	PValue
Diet	26.262	0.00000
Genotype	3.119	0.00076
Diet*Genotype	1.104	0.07874

Summary of Fit

RSquare	0.812233
RSquare Adj	0.799193

Root Mean Square Error	4.483664
Mean of Response	16.69744
Observations (or Sum Wgts)	78

Level	- Level	Difference	Std Err Dif	Lower CL	Upper CL	p-Value
HFD,KO	LFD,KOTG	22.80909	1.911841	17.2115	28.40671	<.0001*
HFD,KO	LFD,KO	20.66485	1.779827	15.4538	25.87594	<.0001*
HFD,KO	LFD,WT	20.40280	1.836837	15.0248	25.78081	<.0001*
HFD,WT	LFD,KOTG	19.37216	1.756138	14.2304	24.51390	<.0001*
HFD,WT	LFD,KO	17.22792	1.611418	12.5099	21.94593	<.0001*
HFD,WT	LFD,WT	16.96587	1.674173	12.0641	21.86762	<.0001*
HFD,KOTG	LFD,KOTG	14.84924	1.871587	9.3695	20.32900	<.0001*
HFD,KOTG	LFD,KO	12.70500	1.736515	7.6207	17.78929	<.0001*
HFD,KOTG	LFD,WT	12.44295	1.794902	7.1877	17.69818	<.0001*
HFD,KO	HFD,KOTG	7.95985	1.871587	2.4801	13.43960	0.0009*
HFD,WT	HFD,KOTG	4.52292	1.712227	-0.4903	9.53609	0.1005
HFD,KO	HFD,WT	3.43693	1.756138	-1.7048	8.57867	0.3769
LFD,WT	LFD,KOTG	2.40629	1.836837	-2.9717	7.78431	0.7786
LFD,KO	LFD,KOTG	2.14424	1.779827	-3.0669	7.35534	0.8332
LFD,WT	LFD,KO	0.26205	1.699005	-4.7124	5.23651	1.0000

Response: Fasted glucose, mg/dl

Source	LogWorth	PValue
Diet	7.838	0.00000
Genotype	5.232	0.00001
Diet*Genotype	0.496	0.31931

Summary of Fit

RSquare	0.475762
RSquare Adj	0.439357
Root Mean Square Error	27.98813
Mean of Response	126.4231
Observations (or Sum Wgts)	78

Level	- Level	Difference	Std Err Dif	Lower CL	Upper CL	p-Value
HFD,KO	LFD,WT	84.25874	11.46599	50.6879	117.8296	<.0001*
HFD,KO	LFD,KOTG	75.72727	11.93418	40.7856	110.6690	<.0001*
HFD,KO	LFD,KO	52.44848	11.11011	19.9196	84.9774	0.0002*
HFD,KO	HFD,KOTG	47.76515	11.68290	13.5592	81.9711	0.0015*
HFD,WT	LFD,WT	42.32692	10.45060	11.7290	72.9249	0.0017*
HFD,KO	HFD,WT	41.93182	10.96224	9.8358	74.0278	0.0036*
HFD,KOTG	LFD,WT	36.49359	11.20422	3.6891	69.2980	0.0205*
HFD,WT	LFD,KOTG	33.79545	10.96224	1.6995	65.8914	0.0332*
LFD,KO	LFD,WT	31.81026	10.60561	0.7585	62.8621	0.0415*
HFD,KOTG	LFD,KOTG	27.96212	11.68290	-6.2439	62.1681	0.1724
LFD,KO	LFD,KOTG	23.27879	11.11011	-9.2501	55.8077	0.3014
HFD,WT	LFD,KO	10.51667	10.05887	-18.9344	39.9677	0.9008
LFD,KOTG	LFD,WT	8.53147	11.46599	-25.0394	42.1023	0.9756

Level	- Level	Difference	Std Err Dif	Lower CL	Upper CL	p-Value
HFD,WT	HFD,KOTG	5.83333	10.68814	-25.4601	37.1268	0.9940
HFD,KOTG	LFD,KO	4.68333	10.83975	-27.0540	36.4207	0.9980

Response: Insulin, ng/ml

Source	LogWorth	PValue
Genotype	8.267	0.00000
Diet	6.602	0.00000
Diet*Genotype	5.535	0.00000

Summary of Fit

RSquare	0.608576
RSquare Adj	0.580212
Root Mean Square Error	1.433345
Mean of Response	1.851551
Observations (or Sum Wgts)	75

Level	- Level	Difference	Std Err Dif	Lower CL	Upper CL	p-Value
HFD,KO	LFD,WT	5.130340	0.5872032	3.40906	6.851622	<.0001*
HFD,KO	HFD,KOTG	5.126553	0.5983121	3.37271	6.880398	<.0001*
HFD,KO	LFD,KOTG	4.949284	0.6442408	3.06081	6.837761	<.0001*
HFD,KO	LFD,KO	4.428213	0.5689781	2.76036	6.096071	<.0001*
HFD,KO	HFD,WT	3.608288	0.5689781	1.94043	5.276146	<.0001*
HFD,WT	LFD,WT	1.522051	0.5431410	-0.07007	3.114172	0.0691
HFD,WT	HFD,KOTG	1.518265	0.5551323	-0.10901	3.145536	0.0812
HFD,WT	LFD,KOTG	1.340995	0.6043515	-0.43055	3.112544	0.2426
HFD,WT	LFD,KO	0.819925	0.5233837	-0.71428	2.354131	0.6229
LFD,KO	LFD,WT	0.702126	0.5431410	-0.88999	2.294248	0.7880
LFD,KO	HFD,KOTG	0.698340	0.5551323	-0.92893	2.325611	0.8064
LFD,KO	LFD,KOTG	0.521070	0.6043515	-1.25048	2.292619	0.9541
LFD,KOTG	LFD,WT	0.181056	0.6215402	-1.64088	2.002991	0.9997
LFD,KOTG	HFD,KOTG	0.177269	0.6320459	-1.67546	2.029999	0.9998
HFD,KOTG	LFD,WT	0.003787	0.5737974	-1.67820	1.685772	1.0000

Response: Raw Data (ITT test)

Level	- Level	Difference	Std Err Dif	Lower CL	Upper CL	p-Value
Time(120)	Time(30)	54.51282	4.662063	41.7344	67.29124	<.0001*
Time(0)	Time(30)	50.14103	4.662063	37.3626	62.91944	<.0001*
Time(90)	Time(30)	42.29487	4.662063	29.5165	55.07329	<.0001*
Time(120)	Time(60)	33.55128	4.662063	20.7729	46.32970	<.0001*
Time(0)	Time(60)	29.17949	4.662063	16.4011	41.95790	<.0001*
Time(90)	Time(60)	21.33333	4.662063	8.5549	34.11175	<.0001*
Time(60)	Time(30)	20.96154	4.662063	8.1831	33.73995	<.0001*
Time(120)	Time(90)	12.21795	4.662063	-0.5605	24.99636	0.0686
Time(0)	Time(90)	7.84615	4.662063	-4.9323	20.62457	0.4457
Time(120)	Time(0)	4.37179	4.662063	-8.4066	17.15021	0.8820

Power

α	σ	δ	Number	Power
0.0500	29.11458	20.37273	390	1.0000

Level	- Level	Difference	Std Err Dif	Lower CL	Upper CL	p-Value
HFD,KO	LFD,WT	90.15385	5.334124	74.8752	105.4325	<.0001*
HFD,KO	LFD,KOTG	82.74545	5.551932	66.8429	98.6480	<.0001*
HFD,KO	LFD,KO	78.21333	5.168567	63.4089	93.0178	<.0001*
HFD,KO	HFD,WT	57.52500	5.099775	42.9176	72.1324	<.0001*
HFD,KO	HFD,KOTG	52.45000	5.435036	36.8823	68.0177	<.0001*
HFD,KOTG	LFD,WT	37.70385	5.212345	22.7740	52.6337	<.0001*
HFD,WT	LFD,WT	32.62885	4.861752	18.7032	46.5545	<.0001*
HFD,KOTG	LFD,KOTG	30.29545	5.435036	14.7277	45.8632	<.0001*
HFD,KOTG	LFD,KO	25.76333	5.042793	11.3191	40.2075	<.0001*
HFD,WT	LFD,KOTG	25.22045	5.099775	10.6130	39.8279	<.0001*
HFD,WT	LFD,KO	20.68833	4.679513	7.2847	34.0920	0.0002*
LFD,KO	LFD,WT	11.94051	4.933864	-2.1917	26.0727	0.1519
LFD,KOTG	LFD,WT	7.40839	5.334124	-7.8703	22.6871	0.7338
HFD,KOTG	HFD,WT	5.07500	4.972260	-9.1672	19.3172	0.9109
LFD,KO	LFD,KOTG	4.53212	5.168567	-10.2724	19.3366	0.9519

Power

α	σ	δ	Number	Power
0.0500	29.11458	10.89908	390	1.0000

Level	- Level	Difference	Std Err Dif	Lower CL	Upper CL	p-Value
[Time(120)]HFD,KO	[Time(30)]LFD,KOTG	148.3636	12.28228	101.940	194.7877	<.0001*
[Time(0)]HFD,KO	[Time(30)]LFD,KOTG	145.7273	12.28228	99.303	192.1514	<.0001*
[Time(120)]HFD,KO	[Time(30)]LFD,KO	144.7515	11.43418	101.533	187.9700	<.0001*
[Time(0)]HFD,KO	[Time(30)]LFD,KO	142.1152	11.43418	98.897	185.3336	<.0001*
[Time(120)]HFD,KO	[Time(60)]LFD,WT	132.5874	11.80043	87.985	177.1902	<.0001*
[Time(0)]HFD,KO	[Time(60)]LFD,WT	129.9510	11.80043	85.348	174.5539	<.0001*
[Time(120)]HFD,KO	[Time(30)]LFD,WT	129.8951	11.80043	85.292	174.4979	<.0001*
[Time(90)]HFD,KO	[Time(30)]LFD,KOTG	129.1818	12.28228	82.758	175.6059	<.0001*
[Time(0)]HFD,KO	[Time(30)]LFD,WT	127.2587	11.80043	82.656	171.8616	<.0001*
[Time(90)]HFD,KO	[Time(30)]LFD,KO	125.5697	11.43418	82.351	168.7882	<.0001*
[Time(120)]HFD,KO	[Time(60)]LFD,KO	124.8182	11.43418	81.600	168.0367	<.0001*
[Time(0)]HFD,KO	[Time(60)]LFD,KO	122.1818	11.43418	78.963	165.4003	<.0001*
[Time(120)]HFD,KO	[Time(30)]HFD,WT	121.9432	11.28199	79.300	164.5864	<.0001*
[Time(0)]HFD,KO	[Time(30)]HFD,WT	119.3068	11.28199	76.664	161.9501	<.0001*
[Time(120)]HFD,KO	[Time(90)]LFD,WT	118.0490	11.80043	73.446	162.6518	<.0001*
[Time(0)]HFD,KO	[Time(90)]LFD,WT	115.4126	11.80043	70.810	160.0154	<.0001*
[Time(120)]HFD,KO	[Time(30)]HFD,KOTG	113.9848	12.02368	68.538	159.4315	<.0001*
[Time(90)]HFD,KO	[Time(60)]LFD,WT	113.4056	11.80043	68.803	158.0084	<.0001*
[Time(0)]HFD,KO	[Time(30)]HFD,KOTG	111.3485	12.02368	65.902	156.7951	<.0001*
[Time(90)]HFD,KO	[Time(30)]LFD,WT	110.7133	11.80043	66.110	155.3161	<.0001*

Level	- Level	Difference	Std Err Dif	Lower CL	Upper CL	p-Value
[Time(120)]HFD,KO	[Time(60)]LFD,KOTG	107.1818	12.28228	60.758	153.6059	<.0001*
[Time(90)]HFD,KO	[Time(60)]LFD,KO	105.6364	11.43418	62.418	148.8548	<.0001*
[Time(0)]HFD,KO	[Time(60)]LFD,KOTG	104.5455	12.28228	58.121	150.9696	<.0001*
[Time(120)]HFD,KO	[Time(0)]LFD,WT	103.1259	11.80043	58.523	147.7287	<.0001*
[Time(90)]HFD,KO	[Time(30)]HFD,WT	102.7614	11.28199	60.118	145.4046	<.0001*
[Time(120)]HFD,KO	[Time(90)]LFD,KOTG	100.5455	12.28228	54.121	146.9696	<.0001*
[Time(0)]HFD,KO	[Time(0)]LFD,WT	100.4895	11.80043	55.887	145.0923	<.0001*
[Time(120)]HFD,KO	[Time(90)]LFD,KO	100.2182	11.43418	57.000	143.4367	<.0001*
[Time(120)]HFD,KO	[Time(120)]LFD,WT	100.2028	11.80043	55.600	144.8056	<.0001*
[Time(60)]HFD,KO	[Time(30)]LFD,KOTG	99.8182	12.28228	53.394	146.2423	<.0001*
[Time(90)]HFD,KO	[Time(90)]LFD,WT	98.8671	11.80043	54.264	143.4700	<.0001*
[Time(120)]HFD,KO	[Time(120)]LFD,KOTG	98.4545	12.28228	52.030	144.8786	<.0001*
[Time(0)]HFD,KO	[Time(90)]LFD,KOTG	97.9091	12.28228	51.485	144.3332	<.0001*
[Time(0)]HFD,KO	[Time(90)]LFD,KO	97.5818	11.43418	54.363	140.8003	<.0001*
[Time(0)]HFD,KO	[Time(120)]LFD,WT	97.5664	11.80043	52.964	142.1693	<.0001*
[Time(120)]HFD,KOTG	[Time(30)]LFD,KOTG	96.2121	12.02368	50.765	141.6588	<.0001*
[Time(60)]HFD,KO	[Time(30)]LFD,KO	96.2061	11.43418	52.988	139.4245	<.0001*
[Time(0)]HFD,KO	[Time(120)]LFD,KOTG	95.8182	12.28228	49.394	142.2423	<.0001*
[Time(120)]HFD,KO	[Time(60)]HFD,WT	95.1932	11.28199	52.550	137.8364	<.0001*
[Time(90)]HFD,KO	[Time(30)]HFD,KOTG	94.8030	12.02368	49.356	140.2497	<.0001*
[Time(120)]HFD,KOTG	[Time(30)]LFD,KO	92.6000	11.15594	50.433	134.7668	<.0001*
[Time(0)]HFD,KO	[Time(60)]HFD,WT	92.5568	11.28199	49.914	135.2001	<.0001*
[Time(120)]HFD,KO	[Time(0)]LFD,KOTG	92.2727	12.28228	45.849	138.6968	<.0001*
[Time(0)]HFD,KO	[Time(0)]LFD,KOTG	89.6364	12.28228	43.212	136.0605	<.0001*
[Time(90)]HFD,KO	[Time(60)]LFD,KOTG	88.0000	12.28228	41.576	134.4241	<.0001*
[Time(90)]HFD,WT	[Time(30)]LFD,KOTG	86.8580	11.28199	44.215	129.5012	<.0001*
[Time(120)]HFD,KO	[Time(60)]HFD,KOTG	86.1515	12.02368	40.705	131.5982	<.0001*
[Time(30)]HFD,KO	[Time(30)]LFD,KOTG	85.6364	12.28228	39.212	132.0605	<.0001*
[Time(60)]HFD,KO	[Time(60)]LFD,WT	84.0420	11.80043	39.439	128.6448	<.0001*
[Time(90)]HFD,KO	[Time(0)]LFD,WT	83.9441	11.80043	39.341	128.5469	<.0001*
[Time(0)]HFD,KO	[Time(60)]HFD,KOTG	83.5152	12.02368	38.069	128.9618	<.0001*
[Time(90)]HFD,WT	[Time(30)]LFD,KO	83.2458	10.35227	44.117	122.3750	<.0001*
[Time(30)]HFD,KO	[Time(30)]LFD,KO	82.0242	11.43418	38.806	125.2427	<.0001*
[Time(90)]HFD,KO	[Time(90)]LFD,KOTG	81.3636	12.28228	34.940	127.7877	<.0001*
[Time(60)]HFD,KO	[Time(30)]LFD,WT	81.3497	11.80043	36.747	125.9525	<.0001*
[Time(90)]HFD,KO	[Time(90)]LFD,KO	81.0364	11.43418	37.818	124.2548	<.0001*
[Time(90)]HFD,KO	[Time(120)]LFD,WT	81.0210	11.80043	36.418	125.6238	<.0001*
[Time(120)]HFD,KOTG	[Time(60)]LFD,WT	80.4359	11.53103	36.851	124.0204	<.0001*
[Time(120)]HFD,WT	[Time(30)]LFD,KOTG	80.4205	11.28199	37.777	123.0637	<.0001*
[Time(90)]HFD,KO	[Time(120)]LFD,KOTG	79.2727	12.28228	32.849	125.6968	<.0001*
[Time(120)]HFD,KOTG	[Time(30)]LFD,WT	77.7436	11.53103	34.159	121.3281	<.0001*
[Time(120)]HFD,KO	[Time(0)]LFD,KO	77.6848	11.43418	34.466	120.9033	<.0001*
[Time(0)]HFD,KOTG	[Time(30)]LFD,KOTG	77.2121	12.02368	31.765	122.6588	<.0001*
[Time(120)]HFD,WT	[Time(30)]LFD,KO	76.8083	10.35227	37.679	115.9375	<.0001*
[Time(120)]HFD,KO	[Time(120)]LFD,KO	76.6848	11.43418	33.466	119.9033	<.0001*
[Time(90)]HFD,KOTG	[Time(30)]LFD,KOTG	76.4621	12.02368	31.015	121.9088	<.0001*
[Time(60)]HFD,KO	[Time(60)]LFD,KO	76.2727	11.43418	33.054	119.4912	<.0001*
[Time(90)]HFD,KO	[Time(60)]HFD,WT	76.0114	11.28199	33.368	118.6546	<.0001*
[Time(0)]HFD,KO	[Time(0)]LFD,KO	75.0485	11.43418	31.830	118.2670	<.0001*
[Time(0)]HFD,WT	[Time(30)]LFD,KOTG	74.2330	11.28199	31.590	116.8762	<.0001*

Level	- Level	Difference	Std Err Dif	Lower CL	Upper CL	p-Value
[Time(120)]HFD,KO	[Time(0)]HFD,WT	74.1307	11.28199	31.487	116.7739	<.0001*
[Time(0)]HFD,KO	[Time(120)]LFD,KO	74.0485	11.43418	30.830	117.2670	<.0001*
[Time(0)]HFD,KOTG	[Time(30)]LFD,KO	73.6000	11.15594	31.433	115.7668	<.0001*
[Time(60)]HFD,KO	[Time(30)]HFD,WT	73.3977	11.28199	30.754	116.0410	<.0001*
[Time(90)]HFD,KO	[Time(0)]LFD,KOTG	73.0909	12.28228	26.667	119.5150	<.0001*
[Time(90)]HFD,KOTG	[Time(30)]LFD,KO	72.8500	11.15594	30.683	115.0168	<.0001*
[Time(120)]HFD,KOTG	[Time(60)]LFD,KO	72.6667	11.15594	30.500	114.8335	<.0001*
[Time(120)]HFD,KO	[Time(90)]HFD,KOTG	71.9015	12.02368	26.455	117.3482	<.0001*
[Time(120)]LFD,KO	[Time(30)]LFD,KOTG	71.6788	11.43418	28.460	114.8973	<.0001*
[Time(0)]HFD,KO	[Time(0)]HFD,WT	71.4943	11.28199	28.851	114.1376	<.0001*
[Time(120)]HFD,KO	[Time(0)]HFD,KOTG	71.1515	12.02368	25.705	116.5982	<.0001*
[Time(90)]HFD,WT	[Time(60)]LFD,WT	71.0817	10.75543	30.429	111.7347	<.0001*
[Time(0)]LFD,KO	[Time(30)]LFD,KOTG	70.6788	11.43418	27.460	113.8973	<.0001*
[Time(0)]HFD,WT	[Time(30)]LFD,KO	70.6208	10.35227	31.492	109.7500	<.0001*
[Time(30)]HFD,KO	[Time(60)]LFD,WT	69.8601	11.80043	25.257	114.4630	<.0001*
[Time(120)]HFD,KOTG	[Time(30)]HFD,WT	69.7917	10.99990	28.215	111.3687	<.0001*
[Time(60)]HFD,KO	[Time(90)]LFD,WT	69.5035	11.80043	24.901	114.1063	<.0001*
[Time(0)]HFD,KO	[Time(90)]HFD,KOTG	69.2652	12.02368	23.819	114.7118	<.0001*
[Time(0)]HFD,KO	[Time(0)]HFD,KOTG	68.5152	12.02368	23.069	113.9618	<.0001*
[Time(90)]HFD,WT	[Time(30)]LFD,WT	68.3894	10.75543	27.736	109.0424	<.0001*
[Time(120)]LFD,KO	[Time(30)]LFD,KO	68.0667	10.51792	28.311	107.8219	<.0001*
[Time(120)]HFD,KO	[Time(120)]HFD,WT	67.9432	11.28199	25.300	110.5864	<.0001*
[Time(30)]HFD,KO	[Time(30)]LFD,WT	67.1678	11.80043	22.565	111.7707	<.0001*
[Time(0)]LFD,KO	[Time(30)]LFD,KO	67.0667	10.51792	27.311	106.8219	<.0001*
[Time(90)]HFD,KO	[Time(60)]HFD,KOTG	66.9697	12.02368	21.523	112.4163	<.0001*
[Time(120)]HFD,KOTG	[Time(90)]LFD,WT	65.8974	11.53103	22.313	109.4820	<.0001*
[Time(60)]HFD,KO	[Time(30)]HFD,KOTG	65.4394	12.02368	19.993	110.8860	<.0001*
[Time(0)]HFD,KO	[Time(120)]HFD,WT	65.3068	11.28199	22.664	107.9501	<.0001*
[Time(120)]HFD,WT	[Time(60)]LFD,WT	64.6442	10.75543	23.991	105.2972	<.0001*
[Time(90)]HFD,WT	[Time(60)]LFD,KO	63.3125	10.35227	24.183	102.4416	<.0001*
[Time(120)]HFD,KO	[Time(30)]HFD,KO	62.7273	12.28228	16.303	109.1514	0.0002*
[Time(60)]HFD,KOTG	[Time(30)]LFD,KOTG	62.2121	12.02368	16.765	107.6588	0.0002*
[Time(30)]HFD,KO	[Time(60)]LFD,KO	62.0909	11.43418	18.872	105.3094	<.0001*
[Time(120)]HFD,WT	[Time(30)]LFD,WT	61.9519	10.75543	21.299	102.6049	<.0001*
[Time(120)]HFD,KOTG	[Time(30)]HFD,KOTG	61.8333	11.75939	17.386	106.2810	0.0001*
[Time(120)]HFD,KO	[Time(90)]HFD,WT	61.5057	11.28199	18.862	104.1489	<.0001*
[Time(0)]HFD,KOTG	[Time(60)]LFD,WT	61.4359	11.53103	17.851	105.0204	<.0001*
[Time(90)]HFD,KOTG	[Time(60)]LFD,WT	60.6859	11.53103	17.101	104.2704	<.0001*
[Time(90)]HFD,WT	[Time(30)]HFD,WT	60.4375	10.18393	21.945	98.9303	<.0001*
[Time(0)]HFD,KO	[Time(30)]HFD,KO	60.0909	12.28228	13.667	106.5150	0.0006*
[Time(30)]HFD,KO	[Time(30)]HFD,WT	59.2159	11.28199	16.573	101.8592	0.0001*
[Time(0)]HFD,KO	[Time(90)]HFD,WT	58.8693	11.28199	16.226	101.5126	0.0001*
[Time(0)]HFD,KOTG	[Time(30)]LFD,WT	58.7436	11.53103	15.159	102.3281	0.0002*
[Time(60)]HFD,KO	[Time(60)]LFD,KOTG	58.6364	12.28228	12.212	105.0605	0.0010*
[Time(60)]HFD,KOTG	[Time(30)]LFD,KO	58.6000	11.15594	16.433	100.7668	0.0001*
[Time(90)]HFD,KO	[Time(0)]LFD,KO	58.5030	11.43418	15.285	101.7215	0.0002*
[Time(0)]HFD,WT	[Time(60)]LFD,WT	58.4567	10.75543	17.804	99.1097	<.0001*
[Time(90)]HFD,KOTG	[Time(30)]LFD,WT	57.9936	11.53103	14.409	101.5781	0.0003*
[Time(90)]HFD,KO	[Time(120)]LFD,KO	57.5030	11.43418	14.285	100.7215	0.0003*
[Time(120)]HFD,WT	[Time(60)]LFD,KO	56.8750	10.35227	17.746	96.0041	<.0001*

Level	- Level	Difference	Std Err Dif	Lower CL	Upper CL	p-Value
[Time(90)]HFD,WT	[Time(90)]LFD,WT	56.5433	10.75543	15.890	97.1962	0.0001*
[Time(0)]LFD,KOTG	[Time(30)]LFD,KOTG	56.0909	12.28228	9.667	102.5150	0.0025*
[Time(120)]LFD,KO	[Time(60)]LFD,WT	55.9026	10.91496	14.647	97.1585	0.0002*
[Time(0)]HFD,WT	[Time(30)]LFD,WT	55.7644	10.75543	15.111	96.4174	0.0001*
[Time(30)]HFD,KO	[Time(90)]LFD,WT	55.3217	11.80043	10.719	99.9245	0.0015*
[Time(120)]HFD,KOTG	[Time(60)]LFD,KOTG	55.0303	12.02368	9.584	100.4769	0.0024*
[Time(90)]HFD,KO	[Time(0)]HFD,WT	54.9489	11.28199	12.306	97.5921	0.0007*
[Time(0)]LFD,KO	[Time(60)]LFD,WT	54.9026	10.91496	13.647	96.1585	0.0003*
[Time(60)]HFD,KO	[Time(0)]LFD,WT	54.5804	11.80043	9.978	99.1833	0.0019*
[Time(120)]HFD,WT	[Time(30)]HFD,WT	54.0000	10.18393	15.507	92.4928	<.0001*
[Time(0)]HFD,KOTG	[Time(60)]LFD,KO	53.6667	11.15594	11.500	95.8335	0.0009*
[Time(120)]LFD,KO	[Time(30)]LFD,WT	53.2103	10.91496	11.954	94.4662	0.0006*
[Time(60)]HFD,WT	[Time(30)]LFD,KOTG	53.1705	11.28199	10.527	95.8137	0.0013*
[Time(90)]HFD,KOTG	[Time(60)]LFD,KO	52.9167	11.15594	10.750	95.0835	0.0012*
[Time(90)]HFD,KO	[Time(90)]HFD,KOTG	52.7197	12.02368	7.273	98.1663	0.0054*
[Time(90)]HFD,WT	[Time(30)]HFD,KOTG	52.4792	10.99990	10.902	94.0562	0.0010*
[Time(0)]LFD,KOTG	[Time(30)]LFD,KO	52.4788	11.43418	9.260	95.6973	0.0023*
[Time(0)]LFD,KO	[Time(30)]LFD,WT	52.2103	10.91496	10.954	93.4662	0.0010*
[Time(120)]HFD,KO	[Time(120)]HFD,KOTG	52.1515	12.02368	6.705	97.5982	0.0065*
[Time(60)]HFD,KO	[Time(90)]LFD,KOTG	52.0000	12.28228	5.576	98.4241	0.0098*
[Time(90)]HFD,KO	[Time(0)]HFD,KOTG	51.9697	12.02368	6.523	97.4163	0.0069*
[Time(60)]HFD,KO	[Time(90)]LFD,KO	51.6727	11.43418	8.454	94.8912	0.0031*
[Time(60)]HFD,KO	[Time(120)]LFD,WT	51.6573	11.80043	7.055	96.2602	0.0055*
[Time(30)]HFD,KO	[Time(30)]HFD,KOTG	51.2576	12.02368	5.811	96.7042	0.0087*
[Time(120)]HFD,KOTG	[Time(0)]LFD,WT	50.9744	11.53103	7.390	94.5589	0.0046*
[Time(0)]HFD,KOTG	[Time(30)]HFD,WT	50.7917	10.99990	9.215	92.3687	0.0020*
[Time(0)]HFD,WT	[Time(60)]LFD,KO	50.6875	10.35227	11.558	89.8166	0.0006*
[Time(120)]HFD,WT	[Time(90)]LFD,WT	50.1058	10.75543	9.453	90.7587	0.0017*
[Time(90)]HFD,KOTG	[Time(30)]HFD,WT	50.0417	10.99990	8.465	91.6187	0.0027*
[Time(60)]HFD,KO	[Time(120)]LFD,KOTG	49.9091	12.28228	3.485	96.3332	0.0186*
[Time(120)]LFD,KOTG	[Time(30)]LFD,KOTG	49.9091	12.28228	3.485	96.3332	0.0186*
[Time(60)]HFD,WT	[Time(30)]LFD,KO	49.5583	10.35227	10.429	88.6875	0.0010*
[Time(0)]HFD,KO	[Time(120)]HFD,KOTG	49.5152	12.02368	4.069	94.9618	0.0152*
[Time(90)]HFD,KO	[Time(120)]HFD,WT	48.7614	11.28199	6.118	91.4046	0.0069*
[Time(120)]HFD,KO	[Time(60)]HFD,KO	48.5455	12.28228	2.121	94.9696	0.0278*
[Time(120)]HFD,KOTG	[Time(90)]LFD,KOTG	48.3939	12.02368	2.947	93.8406	0.0214*
[Time(120)]LFD,WT	[Time(30)]LFD,KOTG	48.1608	11.80043	3.558	92.7637	0.0174*
[Time(90)]LFD,KO	[Time(30)]LFD,KOTG	48.1455	11.43418	4.927	91.3639	0.0107*
[Time(120)]LFD,KO	[Time(60)]LFD,KO	48.1333	10.51792	8.378	87.8886	0.0024*
[Time(120)]HFD,KOTG	[Time(90)]LFD,KO	48.0667	11.15594	5.900	90.2335	0.0073*
[Time(120)]HFD,KOTG	[Time(120)]LFD,WT	48.0513	11.53103	4.467	91.6358	0.0126*
[Time(90)]LFD,KOTG	[Time(30)]LFD,KOTG	47.8182	12.28228	1.394	94.2423	0.0341*
[Time(0)]HFD,WT	[Time(30)]HFD,WT	47.8125	10.18393	9.320	86.3053	0.0014*
[Time(0)]LFD,KO	[Time(60)]LFD,KO	47.1333	10.51792	7.378	86.8886	0.0036*
[Time(0)]HFD,KOTG	[Time(90)]LFD,WT	46.8974	11.53103	3.313	90.4820	0.0184*
[Time(60)]HFD,KO	[Time(60)]HFD,WT	46.6477	11.28199	4.004	89.2910	0.0143*
[Time(60)]HFD,KOTG	[Time(60)]LFD,WT	46.4359	11.53103	2.851	90.0204	0.0212*
[Time(120)]HFD,KOTG	[Time(120)]LFD,KOTG	46.3030	12.02368	0.856	91.7497	0.0394*
[Time(120)]LFD,KOTG	[Time(30)]LFD,KO	46.2970	11.43418	3.078	89.5155	0.0196*
[Time(90)]HFD,KOTG	[Time(90)]LFD,WT	46.1474	11.53103	2.563	89.7320	0.0233*

Level	- Level	Difference	Std Err Dif	Lower CL	Upper CL	p-Value
[Time(120)]HFD,WT	[Time(30)]HFD,KOTG	46.0417	10.99990	4.465	87.6187	0.0118*
[Time(0)]HFD,KO	[Time(60)]HFD,KO	45.9091	12.28228	-0.515	92.3332	0.0573
[Time(90)]HFD,WT	[Time(60)]LFD,KOTG	45.6761	11.28199	3.033	88.3194	0.0196*
[Time(120)]LFD,KO	[Time(30)]HFD,WT	45.2583	10.35227	6.129	84.3875	0.0056*
[Time(0)]LFD,WT	[Time(30)]LFD,KOTG	45.2378	11.80043	0.635	89.8406	0.0418*
[Time(120)]LFD,WT	[Time(30)]LFD,KO	44.5487	10.91496	3.293	85.8047	0.0174*
[Time(90)]LFD,KO	[Time(30)]LFD,KO	44.5333	10.51792	4.778	84.2886	0.0098*
[Time(30)]HFD,KO	[Time(60)]LFD,KOTG	44.4545	12.28228	-1.970	90.8786	0.0830
[Time(0)]LFD,KO	[Time(30)]HFD,WT	44.2583	10.35227	5.129	83.3875	0.0083*
[Time(90)]LFD,KOTG	[Time(30)]LFD,KO	44.2061	11.43418	0.988	87.4245	0.0374*
[Time(0)]HFD,WT	[Time(90)]LFD,WT	43.9183	10.75543	3.265	84.5712	0.0173*
[Time(60)]HFD,KOTG	[Time(30)]LFD,WT	43.7436	11.53103	0.159	87.3281	0.0478*
[Time(60)]HFD,KO	[Time(0)]LFD,KOTG	43.7273	12.28228	-2.697	90.1514	0.0991
[Time(90)]HFD,KO	[Time(30)]HFD,KO	43.5455	12.28228	-2.879	89.9696	0.1035
[Time(120)]HFD,KOTG	[Time(60)]HFD,WT	43.0417	10.99990	1.465	84.6187	0.0319*
[Time(0)]HFD,KOTG	[Time(30)]HFD,KOTG	42.8333	11.75939	-1.614	87.2810	0.0774
[Time(90)]HFD,KO	[Time(90)]HFD,WT	42.3239	11.28199	-0.319	84.9671	0.0548
[Time(90)]HFD,KOTG	[Time(30)]HFD,KOTG	42.0833	11.75939	-2.364	86.5310	0.0938
[Time(0)]LFD,WT	[Time(30)]LFD,KO	41.6256	10.91496	0.370	82.8816	0.0447*
[Time(90)]HFD,WT	[Time(0)]LFD,WT	41.6202	10.75543	0.967	82.2732	0.0370*
[Time(120)]LFD,KO	[Time(90)]LFD,WT	41.3641	10.91496	0.108	82.6200	0.0484*
[Time(60)]LFD,KOTG	[Time(30)]LFD,KOTG	41.1818	12.28228	-5.242	87.6059	0.1759
[Time(30)]HFD,KO	[Time(0)]LFD,WT	40.3986	11.80043	-4.204	85.0014	0.1459
[Time(0)]LFD,KO	[Time(90)]LFD,WT	40.3641	10.91496	-0.892	81.6200	0.0651
[Time(0)]LFD,KOTG	[Time(60)]LFD,WT	40.3147	11.80043	-4.288	84.9175	0.1487
[Time(120)]HFD,KOTG	[Time(0)]LFD,KOTG	40.1212	12.02368	-5.325	85.5679	0.1833
[Time(0)]HFD,WT	[Time(30)]HFD,KOTG	39.8542	10.99990	-1.723	81.4312	0.0821
[Time(120)]HFD,WT	[Time(60)]LFD,KOTG	39.2386	11.28199	-3.405	81.8819	0.1255
[Time(90)]HFD,WT	[Time(90)]LFD,KOTG	39.0398	11.28199	-3.603	81.6830	0.1319
[Time(90)]HFD,WT	[Time(90)]LFD,KO	38.7125	10.35227	-0.417	77.8416	0.0570
[Time(90)]HFD,WT	[Time(120)]LFD,WT	38.6971	10.75543	-1.956	79.3501	0.0886
[Time(60)]HFD,KOTG	[Time(60)]LFD,KO	38.6667	11.15594	-3.500	80.8335	0.1298
[Time(30)]HFD,KO	[Time(90)]LFD,KOTG	37.8182	12.28228	-8.606	84.2423	0.3331
[Time(0)]LFD,KOTG	[Time(30)]LFD,WT	37.6224	11.80043	-6.980	82.2252	0.2627
[Time(60)]HFD,KO	[Time(60)]HFD,KOTG	37.6061	12.02368	-7.841	83.0527	0.3006
[Time(60)]LFD,KOTG	[Time(30)]LFD,KO	37.5697	11.43418	-5.649	80.7882	0.2084
[Time(30)]HFD,KO	[Time(90)]LFD,KO	37.4909	11.43418	-5.728	80.7094	0.2120
[Time(30)]HFD,KO	[Time(120)]LFD,WT	37.4755	11.80043	-7.127	82.0784	0.2703
[Time(60)]HFD,WT	[Time(60)]LFD,WT	37.3942	10.75543	-3.259	78.0472	0.1260
[Time(120)]LFD,KO	[Time(30)]HFD,KOTG	37.3000	11.15594	-4.867	79.4668	0.1802
[Time(90)]HFD,WT	[Time(120)]LFD,KOTG	36.9489	11.28199	-5.694	79.5921	0.2140
[Time(0)]LFD,KO	[Time(30)]HFD,KOTG	36.3000	11.15594	-5.867	78.4668	0.2253
[Time(0)]HFD,KOTG	[Time(60)]LFD,KOTG	36.0303	12.02368	-9.416	81.4769	0.3923
[Time(60)]HFD,KOTG	[Time(30)]HFD,WT	35.7917	10.99990	-5.785	77.3687	0.2253
[Time(30)]HFD,KO	[Time(120)]LFD,KOTG	35.7273	12.28228	-10.697	82.1514	0.4598
[Time(90)]HFD,KOTG	[Time(60)]LFD,KOTG	35.2803	12.02368	-10.166	80.7269	0.4399
[Time(120)]HFD,WT	[Time(0)]LFD,WT	35.1827	10.75543	-5.470	75.8357	0.2160
[Time(60)]HFD,WT	[Time(30)]LFD,WT	34.7019	10.75543	-5.951	75.3549	0.2404
[Time(30)]HFD,KOTG	[Time(30)]LFD,KOTG	34.3788	12.02368	-11.068	79.8254	0.4993
[Time(120)]LFD,KOTG	[Time(60)]LFD,WT	34.1329	11.80043	-10.470	78.7357	0.4727

Level	- Level	Difference	Std Err Dif	Lower CL	Upper CL	p-Value
[Time(120)]HFD,KOTG	[Time(60)]HFD,KOTG	34.0000	11.75939	-10.448	78.4477	0.4737
[Time(90)]HFD,WT	[Time(60)]HFD,WT	33.6875	10.18393	-4.805	72.1803	0.1973
[Time(0)]HFD,WT	[Time(60)]LFD,KOTG	33.0511	11.28199	-9.592	75.6944	0.4435
[Time(90)]HFD,KO	[Time(120)]HFD,KOTG	32.9697	12.02368	-12.477	78.4163	0.5945
[Time(120)]HFD,WT	[Time(90)]LFD,KOTG	32.6023	11.28199	-10.041	75.2455	0.4749
[Time(0)]LFD,KOTG	[Time(60)]LFD,KO	32.5455	11.43418	-10.673	75.7639	0.5098
[Time(30)]HFD,KO	[Time(60)]HFD,WT	32.4659	11.28199	-10.177	75.1092	0.4846
[Time(120)]LFD,WT	[Time(60)]LFD,WT	32.3846	11.29805	-10.319	75.0886	0.4936
[Time(90)]LFD,KO	[Time(60)]LFD,WT	32.3692	10.91496	-8.887	73.6252	0.4157
[Time(120)]HFD,WT	[Time(90)]LFD,KO	32.2750	10.35227	-6.854	71.4041	0.3071
[Time(120)]HFD,WT	[Time(120)]LFD,WT	32.2596	10.75543	-8.393	72.9126	0.3902
[Time(90)]LFD,KOTG	[Time(60)]LFD,WT	32.0420	11.80043	-12.561	76.6448	0.6161
[Time(0)]HFD,KOTG	[Time(0)]LFD,WT	31.9744	11.53103	-11.610	75.5589	0.5695
[Time(60)]HFD,KOTG	[Time(90)]LFD,WT	31.8974	11.53103	-11.687	75.4820	0.5749
[Time(120)]LFD,KOTG	[Time(30)]LFD,WT	31.4406	11.80043	-13.162	76.0434	0.6568
[Time(90)]HFD,KOTG	[Time(0)]LFD,WT	31.2244	11.53103	-12.360	74.8089	0.6221
[Time(90)]HFD,WT	[Time(0)]LFD,KOTG	30.7670	11.28199	-11.876	73.4103	0.6066
[Time(30)]HFD,KOTG	[Time(30)]LFD,KO	30.7667	11.15594	-11.400	72.9335	0.5817
[Time(120)]HFD,WT	[Time(120)]LFD,KOTG	30.5114	11.28199	-12.132	73.1546	0.6249
[Time(120)]LFD,KO	[Time(60)]LFD,KOTG	30.4970	11.43418	-12.722	73.7155	0.6546
[Time(90)]LFD,WT	[Time(30)]LFD,KOTG	30.3147	11.80043	-14.288	74.9175	0.7295
[Time(120)]LFD,WT	[Time(30)]LFD,WT	29.6923	11.29805	-13.012	72.3963	0.6851
[Time(90)]LFD,KO	[Time(30)]LFD,WT	29.6769	10.91496	-11.579	70.9329	0.6132
[Time(0)]LFD,KOTG	[Time(30)]HFD,WT	29.6705	11.28199	-12.973	72.3137	0.6837
[Time(60)]HFD,WT	[Time(60)]LFD,KO	29.6250	10.35227	-9.504	68.7541	0.4974
[Time(30)]HFD,KO	[Time(0)]LFD,KOTG	29.5455	12.28228	-16.879	75.9696	0.8371
[Time(0)]LFD,KO	[Time(60)]LFD,KOTG	29.4970	11.43418	-13.722	72.7155	0.7216
[Time(0)]LFD,WT	[Time(60)]LFD,WT	29.4615	11.29805	-13.242	72.1655	0.7007
[Time(0)]HFD,KOTG	[Time(90)]LFD,KOTG	29.3939	12.02368	-16.053	74.8406	0.8137
[Time(90)]HFD,KO	[Time(60)]HFD,KO	29.3636	12.28228	-17.060	75.7877	0.8455
[Time(90)]LFD,KOTG	[Time(30)]LFD,WT	29.3497	11.80043	-15.253	73.9525	0.7866
[Time(60)]HFD,KO	[Time(0)]LFD,KO	29.1394	11.43418	-14.079	72.3579	0.7444
[Time(0)]HFD,KOTG	[Time(90)]LFD,KO	29.0667	11.15594	-13.100	71.2335	0.7023
[Time(0)]HFD,KOTG	[Time(120)]LFD,WT	29.0513	11.53103	-14.533	72.6358	0.7648
[Time(0)]HFD,WT	[Time(0)]LFD,WT	28.9952	10.75543	-11.658	69.6482	0.6318
[Time(90)]HFD,KOTG	[Time(90)]LFD,KOTG	28.6439	12.02368	-16.803	74.0906	0.8501
[Time(90)]HFD,KOTG	[Time(90)]LFD,KO	28.3167	11.15594	-13.850	70.4835	0.7516
[Time(90)]HFD,KOTG	[Time(120)]LFD,WT	28.3013	11.53103	-15.283	71.8858	0.8077
[Time(60)]HFD,KO	[Time(120)]LFD,KO	28.1394	11.43418	-15.079	71.3579	0.8035
[Time(60)]HFD,KOTG	[Time(30)]HFD,KOTG	27.8333	11.75939	-16.614	72.2810	0.8584
[Time(0)]HFD,KOTG	[Time(120)]LFD,KOTG	27.3030	12.02368	-18.144	72.7497	0.9039
[Time(120)]HFD,WT	[Time(60)]HFD,WT	27.2500	10.18393	-11.243	65.7428	0.6478
[Time(0)]LFD,WT	[Time(30)]LFD,WT	26.7692	11.29805	-15.935	69.4732	0.8571
[Time(60)]HFD,WT	[Time(30)]HFD,WT	26.7500	10.18393	-11.743	65.2428	0.6862
[Time(90)]LFD,WT	[Time(30)]LFD,KO	26.7026	10.91496	-14.553	67.9585	0.8127
[Time(90)]HFD,KOTG	[Time(120)]LFD,KOTG	26.5530	12.02368	-18.894	71.9997	0.9277
[Time(120)]LFD,KO	[Time(0)]LFD,WT	26.4410	10.91496	-14.815	67.6970	0.8272
[Time(30)]HFD,WT	[Time(30)]LFD,KOTG	26.4205	11.28199	-16.223	69.0637	0.8714
[Time(0)]HFD,WT	[Time(90)]LFD,KOTG	26.4148	11.28199	-16.228	69.0580	0.8716
[Time(120)]LFD,KOTG	[Time(60)]LFD,KO	26.3636	11.43418	-16.855	69.5821	0.8887

Level	- Level	Difference	Std Err Dif	Lower CL	Upper CL	p-Value
[Time(0)]HFD,WT	[Time(90)]LFD,KO	26.0875	10.35227	-13.042	65.2166	0.7644
[Time(0)]HFD,WT	[Time(120)]LFD,WT	26.0721	10.75543	-14.581	66.7251	0.8262
[Time(0)]LFD,KOTG	[Time(90)]LFD,WT	25.7762	11.80043	-18.827	70.3791	0.9356
[Time(60)]HFD,KO	[Time(0)]HFD,WT	25.5852	11.28199	-17.058	68.2285	0.9052
[Time(120)]HFD,KOTG	[Time(0)]LFD,KO	25.5333	11.15594	-16.633	67.7001	0.8963
[Time(0)]LFD,KO	[Time(0)]LFD,WT	25.4410	10.91496	-15.815	66.6970	0.8768
[Time(60)]LFD,KOTG	[Time(60)]LFD,WT	25.4056	11.80043	-19.197	70.0084	0.9450
[Time(90)]HFD,WT	[Time(60)]HFD,KOTG	24.6458	10.99990	-16.931	66.2228	0.9160
[Time(120)]LFD,WT	[Time(60)]LFD,KO	24.6154	10.91496	-16.641	65.8713	0.9103
[Time(90)]LFD,KO	[Time(60)]LFD,KO	24.6000	10.51792	-15.155	64.3552	0.8728
[Time(120)]HFD,KOTG	[Time(120)]LFD,KO	24.5333	11.15594	-17.633	66.7001	0.9308
[Time(120)]HFD,WT	[Time(0)]LFD,KOTG	24.3295	11.28199	-18.314	66.9728	0.9440
[Time(0)]HFD,WT	[Time(120)]LFD,KOTG	24.3239	11.28199	-18.319	66.9671	0.9442
[Time(90)]LFD,KOTG	[Time(60)]LFD,KO	24.2727	11.43418	-18.946	67.4912	0.9531
[Time(0)]HFD,KOTG	[Time(60)]HFD,WT	24.0417	10.99990	-17.535	65.6187	0.9352
[Time(120)]LFD,KO	[Time(90)]LFD,KOTG	23.8606	11.43418	-19.358	67.0791	0.9616
[Time(60)]LFD,KO	[Time(30)]LFD,KOTG	23.5455	11.43418	-19.673	66.7639	0.9672
[Time(120)]LFD,KO	[Time(90)]LFD,KO	23.5333	10.51792	-16.222	63.2886	0.9172
[Time(120)]LFD,KO	[Time(120)]LFD,WT	23.5179	10.91496	-17.738	64.7739	0.9445
[Time(120)]LFD,KOTG	[Time(30)]HFD,WT	23.4886	11.28199	-19.155	66.1319	0.9626
[Time(30)]HFD,KO	[Time(60)]HFD,KOTG	23.4242	12.02368	-22.022	68.8709	0.9838
[Time(60)]HFD,KO	[Time(90)]HFD,KOTG	23.3561	12.02368	-22.091	68.8027	0.9845
[Time(90)]HFD,KOTG	[Time(60)]HFD,WT	23.2917	10.99990	-18.285	64.8687	0.9544
[Time(0)]LFD,KO	[Time(90)]LFD,KOTG	22.8606	11.43418	-20.358	66.0791	0.9773
[Time(60)]HFD,WT	[Time(90)]LFD,WT	22.8558	10.75543	-17.797	63.5087	0.9525
[Time(30)]HFD,WT	[Time(30)]LFD,KO	22.8083	10.35227	-16.321	61.9375	0.9294
[Time(60)]LFD,KOTG	[Time(30)]LFD,WT	22.7133	11.80043	-21.890	67.3161	0.9863
[Time(60)]HFD,KO	[Time(0)]HFD,KOTG	22.6061	12.02368	-22.841	68.0527	0.9901
[Time(0)]LFD,KO	[Time(90)]LFD,KO	22.5333	10.51792	-17.222	62.2886	0.9480
[Time(0)]LFD,KO	[Time(120)]LFD,WT	22.5179	10.91496	-18.738	63.7739	0.9665
[Time(120)]HFD,KOTG	[Time(0)]HFD,WT	21.9792	10.99990	-19.598	63.5562	0.9775
[Time(120)]LFD,KO	[Time(120)]LFD,KOTG	21.7697	11.43418	-21.449	64.9882	0.9882
[Time(120)]LFD,WT	[Time(30)]HFD,WT	21.7404	10.75543	-18.913	62.3933	0.9739
[Time(90)]LFD,KO	[Time(30)]HFD,WT	21.7250	10.35227	-17.404	60.8541	0.9589
[Time(0)]LFD,KOTG	[Time(30)]HFD,KOTG	21.7121	12.02368	-23.735	67.1588	0.9945
[Time(0)]LFD,WT	[Time(60)]LFD,KO	21.6923	10.91496	-19.564	62.9483	0.9790
[Time(90)]LFD,KOTG	[Time(30)]HFD,WT	21.3977	11.28199	-21.246	64.0410	0.9888
[Time(0)]HFD,KOTG	[Time(0)]LFD,KOTG	21.1212	12.02368	-24.325	66.5679	0.9964
[Time(0)]HFD,WT	[Time(60)]HFD,WT	21.0625	10.18393	-17.430	59.5553	0.9655
[Time(60)]HFD,KOTG	[Time(60)]LFD,KOTG	21.0303	12.02368	-24.416	66.4769	0.9966
[Time(0)]LFD,KO	[Time(120)]LFD,KOTG	20.7697	11.43418	-22.449	63.9882	0.9940
[Time(90)]HFD,KOTG	[Time(0)]LFD,KOTG	20.3712	12.02368	-25.075	65.8179	0.9980
[Time(60)]LFD,KO	[Time(30)]LFD,KO	19.9333	10.51792	-19.822	59.6886	0.9889
[Time(120)]HFD,KOTG	[Time(90)]HFD,KOTG	19.7500	11.75939	-24.698	64.1977	0.9982
[Time(120)]LFD,KOTG	[Time(90)]LFD,WT	19.5944	11.80043	-25.008	64.1972	0.9985
[Time(60)]HFD,KO	[Time(120)]HFD,WT	19.3977	11.28199	-23.246	62.0410	0.9974
[Time(120)]HFD,KO	[Time(90)]HFD,KO	19.1818	12.28228	-27.242	65.6059	0.9995
[Time(120)]HFD,KOTG	[Time(0)]HFD,KOTG	19.0000	11.75939	-25.448	63.4477	0.9991
[Time(0)]LFD,WT	[Time(30)]HFD,WT	18.8173	10.75543	-21.836	59.4703	0.9966
[Time(60)]HFD,WT	[Time(30)]HFD,KOTG	18.7917	10.99990	-22.785	60.3687	0.9977

Level	- Level	Difference	Std Err Dif	Lower CL	Upper CL	p-Value
[Time(30)]HFD,KOTG	[Time(60)]LFD,WT	18.6026	11.53103	-24.982	62.1871	0.9991
[Time(120)]LFD,KO	[Time(60)]HFD,WT	18.5083	10.35227	-20.621	57.6375	0.9953
[Time(30)]LFD,WT	[Time(30)]LFD,KOTG	18.4685	11.80043	-26.134	63.0714	0.9995
[Time(120)]HFD,WT	[Time(60)]HFD,KOTG	18.2083	10.99990	-23.369	59.7853	0.9986
[Time(0)]HFD,WT	[Time(0)]LFD,KOTG	18.1420	11.28199	-24.501	60.7853	0.9992
[Time(120)]LFD,WT	[Time(90)]LFD,WT	17.8462	11.29805	-24.858	60.5501	0.9994
[Time(90)]LFD,KO	[Time(90)]LFD,WT	17.8308	10.91496	-23.425	59.0867	0.9989
[Time(60)]LFD,KOTG	[Time(60)]LFD,KO	17.6364	11.43418	-25.582	60.8548	0.9996
[Time(0)]LFD,KO	[Time(60)]HFD,WT	17.5083	10.35227	-21.621	56.6375	0.9980
[Time(90)]LFD,KOTG	[Time(90)]LFD,WT	17.5035	11.80043	-27.099	62.1063	0.9998
[Time(60)]HFD,KOTG	[Time(0)]LFD,WT	16.9744	11.53103	-26.610	60.5589	0.9998
[Time(0)]HFD,KO	[Time(90)]HFD,KO	16.5455	12.28228	-29.879	62.9696	1.0000
[Time(90)]HFD,WT	[Time(0)]LFD,KO	16.1792	10.35227	-22.950	55.3083	0.9995
[Time(30)]HFD,KOTG	[Time(30)]LFD,WT	15.9103	11.53103	-27.674	59.4948	1.0000
[Time(120)]HFD,KOTG	[Time(120)]HFD,WT	15.7917	10.99990	-25.785	57.3687	0.9999
[Time(60)]LFD,WT	[Time(30)]LFD,KOTG	15.7762	11.80043	-28.827	60.3791	1.0000
[Time(120)]LFD,KO	[Time(0)]LFD,KOTG	15.5879	11.43418	-27.631	58.8064	1.0000
[Time(120)]LFD,KOTG	[Time(30)]HFD,KOTG	15.5303	12.02368	-29.916	60.9769	1.0000
[Time(90)]HFD,WT	[Time(120)]LFD,KO	15.1792	10.35227	-23.950	54.3083	0.9998
[Time(0)]HFD,KOTG	[Time(60)]HFD,KOTG	15.0000	11.75939	-29.448	59.4477	1.0000
[Time(30)]HFD,KO	[Time(0)]LFD,KO	14.9576	11.43418	-28.261	58.1761	1.0000
[Time(0)]LFD,WT	[Time(90)]LFD,WT	14.9231	11.29805	-27.781	57.6270	1.0000
[Time(0)]LFD,KOTG	[Time(60)]LFD,KOTG	14.9091	12.28228	-31.515	61.3332	1.0000
[Time(30)]LFD,WT	[Time(30)]LFD,KO	14.8564	10.91496	-26.400	56.1124	1.0000
[Time(60)]LFD,KOTG	[Time(30)]HFD,WT	14.7614	11.28199	-27.882	57.4046	1.0000
[Time(0)]LFD,KO	[Time(0)]LFD,KOTG	14.5879	11.43418	-28.631	57.8064	1.0000
[Time(90)]LFD,WT	[Time(60)]LFD,WT	14.5385	11.29805	-28.166	57.2424	1.0000
[Time(60)]HFD,KOTG	[Time(90)]LFD,KOTG	14.3939	12.02368	-31.053	59.8406	1.0000
[Time(90)]HFD,KOTG	[Time(60)]HFD,KOTG	14.2500	11.75939	-30.198	58.6977	1.0000
[Time(60)]HFD,KO	[Time(30)]HFD,KO	14.1818	12.28228	-32.242	60.6059	1.0000
[Time(60)]HFD,KOTG	[Time(90)]LFD,KO	14.0667	11.15594	-28.100	56.2335	1.0000
[Time(60)]HFD,KOTG	[Time(120)]LFD,WT	14.0513	11.53103	-29.533	57.6358	1.0000
[Time(30)]HFD,KO	[Time(120)]LFD,KO	13.9576	11.43418	-29.261	57.1761	1.0000
[Time(120)]LFD,WT	[Time(30)]HFD,KOTG	13.7821	11.53103	-29.802	57.3666	1.0000
[Time(90)]LFD,KO	[Time(30)]HFD,KOTG	13.7667	11.15594	-28.400	55.9335	1.0000
[Time(90)]LFD,KOTG	[Time(30)]HFD,KOTG	13.4394	12.02368	-32.007	58.8860	1.0000
[Time(60)]HFD,KO	[Time(90)]HFD,WT	12.9602	11.28199	-29.683	55.6035	1.0000
[Time(90)]HFD,WT	[Time(0)]HFD,WT	12.6250	10.18393	-25.868	51.1178	1.0000
[Time(60)]HFD,KOTG	[Time(120)]LFD,KOTG	12.3030	12.02368	-33.144	57.7497	1.0000
[Time(60)]LFD,WT	[Time(30)]LFD,KO	12.1641	10.91496	-29.092	53.4200	1.0000
[Time(0)]HFD,WT	[Time(60)]HFD,KOTG	12.0208	10.99990	-29.556	53.5978	1.0000
[Time(60)]HFD,WT	[Time(60)]LFD,KOTG	11.9886	11.28199	-30.655	54.6319	1.0000
[Time(90)]LFD,WT	[Time(30)]LFD,WT	11.8462	11.29805	-30.858	54.5501	1.0000
[Time(30)]HFD,KO	[Time(0)]HFD,WT	11.4034	11.28199	-31.240	54.0467	1.0000
[Time(60)]LFD,KOTG	[Time(90)]LFD,WT	10.8671	11.80043	-33.736	55.4700	1.0000
[Time(0)]LFD,WT	[Time(30)]HFD,KOTG	10.8590	11.53103	-32.726	54.4435	1.0000
[Time(0)]LFD,KOTG	[Time(0)]LFD,WT	10.8531	11.80043	-33.750	55.4560	1.0000
[Time(30)]HFD,KOTG	[Time(60)]LFD,KO	10.8333	11.15594	-31.333	53.0001	1.0000
[Time(30)]HFD,WT	[Time(60)]LFD,WT	10.6442	10.75543	-30.009	51.2972	1.0000
[Time(120)]HFD,KOTG	[Time(30)]HFD,KO	10.5758	12.02368	-34.871	56.0224	1.0000

Level	- Level	Difference	Std Err Dif	Lower CL	Upper CL	p-Value
[Time(90)]HFD,WT	[Time(90)]HFD,KOTG	10.3958	10.99990	-31.181	51.9728	1.0000
[Time(120)]HFD,WT	[Time(0)]LFD,KO	9.7417	10.35227	-29.387	48.8708	1.0000
[Time(90)]HFD,WT	[Time(0)]HFD,KOTG	9.6458	10.99990	-31.931	51.2228	1.0000
[Time(120)]LFD,KO	[Time(60)]HFD,KOTG	9.4667	11.15594	-32.700	51.6335	1.0000
[Time(120)]HFD,KOTG	[Time(90)]HFD,WT	9.3542	10.99990	-32.223	50.9312	1.0000
[Time(30)]HFD,KO	[Time(90)]HFD,KOTG	9.1742	12.02368	-36.272	54.6209	1.0000
[Time(60)]HFD,KOTG	[Time(60)]HFD,WT	9.0417	10.99990	-32.535	50.6187	1.0000
[Time(120)]HFD,WT	[Time(120)]LFD,KO	8.7417	10.35227	-30.387	47.8708	1.0000
[Time(120)]LFD,KOTG	[Time(60)]LFD,KOTG	8.7273	12.28228	-37.697	55.1514	1.0000
[Time(0)]LFD,KO	[Time(60)]HFD,KOTG	8.4667	11.15594	-33.700	50.6335	1.0000
[Time(30)]HFD,KO	[Time(0)]HFD,KOTG	8.4242	12.02368	-37.022	53.8709	1.0000
[Time(0)]LFD,KOTG	[Time(90)]LFD,KOTG	8.2727	12.28228	-38.151	54.6968	1.0000
[Time(30)]HFD,KOTG	[Time(30)]HFD,WT	7.9583	10.99990	-33.619	49.5353	1.0000
[Time(30)]HFD,WT	[Time(30)]LFD,WT	7.9519	10.75543	-32.701	48.6049	1.0000
[Time(0)]LFD,KOTG	[Time(90)]LFD,KO	7.9455	11.43418	-35.273	51.1639	1.0000
[Time(60)]HFD,WT	[Time(0)]LFD,WT	7.9327	10.75543	-32.720	48.5857	1.0000
[Time(0)]LFD,KOTG	[Time(120)]LFD,WT	7.9301	11.80043	-36.673	52.5329	1.0000
[Time(60)]LFD,KO	[Time(60)]LFD,WT	7.7692	10.91496	-33.487	49.0252	1.0000
[Time(120)]LFD,WT	[Time(60)]LFD,KOTG	6.9790	11.80043	-37.624	51.5819	1.0000
[Time(90)]LFD,KO	[Time(60)]LFD,KOTG	6.9636	11.43418	-36.255	50.1821	1.0000
[Time(60)]LFD,KOTG	[Time(30)]HFD,KOTG	6.8030	12.02368	-38.644	52.2497	1.0000
[Time(90)]LFD,WT	[Time(60)]LFD,KO	6.7692	10.91496	-34.487	48.0252	1.0000
[Time(90)]LFD,KOTG	[Time(60)]LFD,KOTG	6.6364	12.28228	-39.788	53.0605	1.0000
[Time(0)]HFD,KOTG	[Time(0)]LFD,KO	6.5333	11.15594	-35.633	48.7001	1.0000
[Time(90)]HFD,WT	[Time(120)]HFD,WT	6.4375	10.18393	-32.055	44.9303	1.0000
[Time(120)]HFD,WT	[Time(0)]HFD,WT	6.1875	10.18393	-32.305	44.6803	1.0000
[Time(0)]LFD,KOTG	[Time(120)]LFD,KOTG	6.1818	12.28228	-40.242	52.6059	1.0000
[Time(60)]HFD,KOTG	[Time(0)]LFD,KOTG	6.1212	12.02368	-39.325	51.5679	1.0000
[Time(90)]HFD,KOTG	[Time(0)]LFD,KO	5.7833	11.15594	-36.383	47.9501	1.0000
[Time(0)]HFD,KOTG	[Time(120)]LFD,KO	5.5333	11.15594	-36.633	47.7001	1.0000
[Time(60)]HFD,WT	[Time(90)]LFD,KOTG	5.3523	11.28199	-37.291	47.9955	1.0000
[Time(30)]HFD,KO	[Time(120)]HFD,WT	5.2159	11.28199	-37.427	47.8592	1.0000
[Time(60)]LFD,KO	[Time(30)]LFD,WT	5.0769	10.91496	-36.179	46.3329	1.0000
[Time(60)]HFD,WT	[Time(90)]LFD,KO	5.0250	10.35227	-34.104	44.1541	1.0000
[Time(60)]HFD,WT	[Time(120)]LFD,WT	5.0096	10.75543	-35.643	45.6626	1.0000
[Time(90)]HFD,KOTG	[Time(120)]LFD,KO	4.7833	11.15594	-37.383	46.9501	1.0000
[Time(120)]LFD,KOTG	[Time(0)]LFD,WT	4.6713	11.80043	-39.932	49.2742	1.0000
[Time(30)]HFD,KOTG	[Time(90)]LFD,WT	4.0641	11.53103	-39.520	47.6487	1.0000
[Time(0)]LFD,WT	[Time(60)]LFD,KOTG	4.0559	11.80043	-40.547	48.6588	1.0000
[Time(120)]HFD,WT	[Time(90)]HFD,KOTG	3.9583	10.99990	-37.619	45.5353	1.0000
[Time(90)]LFD,WT	[Time(30)]HFD,WT	3.8942	10.75543	-36.759	44.5472	1.0000
[Time(30)]LFD,KO	[Time(30)]LFD,KOTG	3.6121	11.43418	-39.606	46.8306	1.0000
[Time(60)]HFD,KO	[Time(120)]HFD,KOTG	3.6061	12.02368	-41.841	49.0527	1.0000
[Time(0)]HFD,WT	[Time(0)]LFD,KO	3.5542	10.35227	-35.575	42.6833	1.0000
[Time(60)]HFD,WT	[Time(120)]LFD,KOTG	3.2614	11.28199	-39.382	45.9046	1.0000
[Time(120)]HFD,WT	[Time(0)]HFD,KOTG	3.2083	10.99990	-38.369	44.7853	1.0000
[Time(0)]HFD,KOTG	[Time(0)]HFD,WT	2.9792	10.99990	-38.598	44.5562	1.0000
[Time(120)]LFD,WT	[Time(0)]LFD,WT	2.9231	11.29805	-39.781	45.6270	1.0000
[Time(0)]LFD,KOTG	[Time(60)]HFD,WT	2.9205	11.28199	-39.723	45.5637	1.0000
[Time(90)]LFD,KO	[Time(0)]LFD,WT	2.9077	10.91496	-38.348	44.1636	1.0000

Level	- Level	Difference	Std Err Dif	Lower CL	Upper CL	p-Value
[Time(30)]HFD,WT	[Time(60)]LFD,KO	2.8750	10.35227	-36.254	42.0041	1.0000
[Time(30)]LFD,WT	[Time(60)]LFD,WT	2.6923	11.29805	-40.012	45.3963	1.0000
[Time(120)]HFD,KO	[Time(0)]HFD,KO	2.6364	12.28228	-43.788	49.0605	1.0000
[Time(90)]LFD,KOTG	[Time(0)]LFD,WT	2.5804	11.80043	-42.022	47.1833	1.0000
[Time(0)]HFD,WT	[Time(120)]LFD,KO	2.5542	10.35227	-36.575	41.6833	1.0000
[Time(90)]HFD,KOTG	[Time(0)]HFD,WT	2.2292	10.99990	-39.348	43.8062	1.0000
[Time(120)]LFD,KOTG	[Time(90)]LFD,KOTG	2.0909	12.28228	-44.333	48.5150	1.0000
[Time(120)]LFD,KOTG	[Time(90)]LFD,KO	1.7636	11.43418	-41.455	44.9821	1.0000
[Time(120)]LFD,KOTG	[Time(120)]LFD,WT	1.7483	11.80043	-42.855	46.3511	1.0000
[Time(90)]HFD,WT	[Time(30)]HFD,KO	1.2216	11.28199	-41.422	43.8649	1.0000
[Time(120)]LFD,KO	[Time(0)]LFD,KO	1.0000	10.51792	-38.755	40.7552	1.0000
[Time(0)]HFD,KOTG	[Time(90)]HFD,KOTG	0.7500	11.75939	-43.698	45.1977	1.0000
[Time(120)]LFD,WT	[Time(90)]LFD,KOTG	0.3427	11.80043	-44.260	44.9455	1.0000
[Time(90)]LFD,KO	[Time(90)]LFD,KOTG	0.3273	11.43418	-42.891	43.5458	1.0000
[Time(120)]LFD,WT	[Time(90)]LFD,KO	0.0154	10.91496	-41.241	41.2713	1.0000

Response: Fpg-sensitive lesion density/10 kb

Source	LogWorth	PValue
Genotype	2.633	0.00233
Diet	1.200	0.06314
Diet*Genotype	0.245	0.56828

Summary of Fit

RSquare	0.372134
RSquare Adj	0.277003
Root Mean Square Error	0.302627
Mean of Response	0.438718
Observations (or Sum Wgts)	39

Level	- Level	Difference	Std Err Dif	Lower CL	Upper CL	p-Value
HFD,KO	LFD,KOTG	0.5632857	0.1617611	0.074195	1.052376	0.0164*
HFD,KO	HFD,KOTG	0.5048036	0.1566245	0.031244	0.978363	0.0312*
HFD,KO	LFD,WT	0.4506286	0.1772004	-0.085143	0.986400	0.1409
HFD,WT	LFD,KOTG	0.4280238	0.1683663	-0.081038	0.937085	0.1412
LFD,KO	LFD,KOTG	0.3716905	0.1683663	-0.137371	0.880752	0.2615
HFD,WT	HFD,KOTG	0.3695417	0.1634374	-0.124617	0.863700	0.2386
HFD,WT	LFD,WT	0.3153667	0.1832499	-0.238696	0.869429	0.5283
LFD,KO	HFD,KOTG	0.3132083	0.1634374	-0.180950	0.807367	0.4106
LFD,KO	LFD,WT	0.2590333	0.1832499	-0.295029	0.813096	0.7188
HFD,KO	LFD,KO	0.1915952	0.1683663	-0.317466	0.700657	0.8619
HFD,KO	HFD,WT	0.1352619	0.1683663	-0.373799	0.644323	0.9649
LFD,WT	LFD,KOTG	0.1126571	0.1772004	-0.423114	0.648429	0.9874
HFD,KOTG	LFD,KOTG	0.0584821	0.1566245	-0.415078	0.532042	0.9990
HFD,WT	LFD,KO	0.0563333	0.1747220	-0.471945	0.584611	0.9995
LFD,WT	HFD,KOTG	0.0541750	0.1725241	-0.467458	0.575808	0.9996

Response for qRT-PCR: *Nd1/28SrRNA*

Source	LogWorth	PValue	
Genotype	4.324	0.00005	
Diet*Genotype	0.998	0.10037	
Diet	0.376	0.42115	^

Summary of Fit

RSquare	0.32284
RSquare Adj	0.264464
Root Mean Square Error	0.567574
Mean of Response	1.030007
Observations (or Sum Wgts)	64

Level	- Level	Difference	Std Err Dif	Lower CL	Upper CL	p-Value
HFD,KO	HFD,WT	1.190054	0.2479911	0.459197	1.920912	0.0002*
HFD,KO	LFD,WT	0.978822	0.2430208	0.262612	1.695032	0.0022*
HFD,KO	LFD,KOTG	0.770330	0.2607824	0.001775	1.538886	0.0491*
HFD,KO	HFD,KOTG	0.733857	0.2538270	-0.014200	1.481914	0.0575
LFD,KO	HFD,WT	0.668525	0.2369189	-0.029702	1.366752	0.0681
HFD,KO	LFD,KO	0.521529	0.2430208	-0.194680	1.237739	0.2789
LFD,KO	LFD,WT	0.457293	0.2317113	-0.225587	1.140172	0.3698
HFD,KOTG	HFD,WT	0.456197	0.2479911	-0.274660	1.187055	0.4492
LFD,KOTG	HFD,WT	0.419724	0.2551057	-0.332101	1.171549	0.5727
LFD,KO	LFD,KOTG	0.248801	0.2502768	-0.488793	0.986395	0.9180
HFD,KOTG	LFD,WT	0.244965	0.2430208	-0.471245	0.961175	0.9134
LFD,KO	HFD,KOTG	0.212328	0.2430208	-0.503882	0.928537	0.9513
LFD,WT	HFD,WT	0.211232	0.2369189	-0.486994	0.909459	0.9470
LFD,KOTG	LFD,WT	0.208491	0.2502768	-0.529102	0.946085	0.9601
HFD,KOTG	LFD,KOTG	0.036473	0.2607824	-0.732082	0.805029	1.0000

Response for qRT-PCR: *Dloop/28SrRNA*

Source	LogWorth	PValue
Genotype	1.398	0.03996
Diet	0.937	0.11549
Diet*Genotype	0.584	0.26090

Summary of Fit

RSquare	0.168078
RSquare Adj	0.09636
Root Mean Square Error	0.643473
Mean of Response	1.206395
Observations (or Sum Wgts)	64

Level	- Level	Difference	Std Err Dif	Lower CL	Upper CL	p-Value
HFD,KO	LFD,KOTG	0.8200144	0.2956554	-0.051315	1.691344	0.0764
HFD,KO	HFD,KOTG	0.6391840	0.2877698	-0.208906	1.487274	0.2441
HFD,KO	LFD,KO	0.6108258	0.2755186	-0.201159	1.422810	0.2459
LFD,WT	LFD,KOTG	0.5858944	0.2837449	-0.250334	1.422122	0.3199
HFD,WT	LFD,KOTG	0.5696526	0.2892195	-0.282710	1.422015	0.3720
LFD,WT	HFD,KOTG	0.4050640	0.2755186	-0.406920	1.217048	0.6842
HFD,WT	HFD,KOTG	0.3888222	0.2811535	-0.439769	1.217413	0.7369
LFD,WT	LFD,KO	0.3767058	0.2626967	-0.397491	1.150903	0.7064
HFD,WT	LFD,KO	0.3604640	0.2686007	-0.431133	1.152061	0.7605
HFD,KO	HFD,WT	0.2503618	0.2811535	-0.578229	1.078953	0.9473
HFD,KO	LFD,WT	0.2341200	0.2755186	-0.577864	1.046104	0.9566
LFD,KO	LFD,KOTG	0.2091886	0.2837449	-0.627039	1.045417	0.9764
HFD,KOTG	LFD,KOTG	0.1808304	0.2956554	-0.690499	1.052160	0.9898
LFD,KO	HFD,KOTG	0.0283582	0.2755186	-0.783626	0.840343	1.0000
LFD,WT	HFD,WT	0.0162418	0.2686007	-0.775355	0.807838	1.0000

Response for Quantitative Reverse Transcription PCR

Response: *Pdk4/28SrRNA*

Source	LogWorth	PValue	
Diet*Genotype	0.896	0.12701	
Diet	0.868	0.13565	^
Genotype	0.393	0.40480	^

Summary of Fit

RSquare	0.214937
RSquare Adj	0.079582
Root Mean Square Error	1.264794
Mean of Response	0.806844
Observations (or Sum Wgts)	35

Level	- Level	Difference	Std Err Dif	Lower CL	Upper CL	p-Value
HFD,KO	LFD,KO	1.900184	0.7658708	-0.43456	4.234928	0.1630
HFD,KO	HFD,KOTG	1.787456	0.7658708	-0.54729	4.122200	0.2134
HFD,KO	LFD,WT	1.645039	0.7658708	-0.68970	3.979783	0.2921
HFD,KO	LFD,KOTG	1.512314	0.7658708	-0.82243	3.847058	0.3804
HFD,KO	HFD,WT	1.296661	0.7658708	-1.03808	3.631405	0.5470
HFD,WT	LFD,KO	0.603523	0.7302291	-1.62257	2.829614	0.9601
HFD,WT	HFD,KOTG	0.490795	0.7302291	-1.73530	2.716886	0.9837
LFD,KOTG	LFD,KO	0.387870	0.7302291	-1.83822	2.613961	0.9944
HFD,WT	LFD,WT	0.348378	0.7302291	-1.87771	2.574469	0.9966
LFD,KOTG	HFD,KOTG	0.275142	0.7302291	-1.95095	2.501233	0.9989
LFD,WT	LFD,KO	0.255145	0.7302291	-1.97095	2.481236	0.9992
HFD,WT	LFD,KOTG	0.215653	0.7302291	-2.01044	2.441744	0.9997
LFD,WT	HFD,KOTG	0.142417	0.7302291	-2.08367	2.368508	1.0000

Level	- Level	Difference	Std Err Dif	Lower CL	Upper CL	p-Value
LFD,KOTG	LFD,WT	0.132725	0.7302291	-2.09337	2.358816	1.0000
HFD,KOTG	LFD,KO	0.112728	0.7302291	-2.11336	2.338819	1.0000

Response: *Glut2/28SrRNA*

Source	LogWorth	PValue	
Diet*Genotype	1.329	0.04686	
Genotype	1.176	0.06663	^
Diet	0.593	0.25532	^

Summary of Fit

RSquare	0.319182
RSquare Adj	0.2018
Root Mean Square Error	0.569666
Mean of Response	0.68724
Observations (or Sum Wgts)	35

Level	- Level	Difference	Std Err Dif	Lower CL	Upper CL	p-Value
HFD,KO	LFD,WT	0.9183070	0.3449500	-0.13327	1.969881	0.1144
HFD,KO	LFD,KO	0.9174120	0.3449500	-0.13416	1.968986	0.1150
HFD,KO	HFD,WT	0.8662870	0.3449500	-0.18529	1.917861	0.1539
LFD,KOTG	LFD,WT	0.7113883	0.3288969	-0.29125	1.714025	0.2851
LFD,KOTG	LFD,KO	0.7104933	0.3288969	-0.29214	1.713130	0.2864
LFD,KOTG	HFD,WT	0.6593683	0.3288969	-0.34327	1.662005	0.3641
HFD,KO	HFD,KOTG	0.5043437	0.3449500	-0.54723	1.555918	0.6899
HFD,KOTG	LFD,WT	0.4139633	0.3288969	-0.58867	1.416600	0.8043
HFD,KOTG	LFD,KO	0.4130683	0.3288969	-0.58957	1.415705	0.8057
HFD,KOTG	HFD,WT	0.3619433	0.3288969	-0.64069	1.364580	0.8772
LFD,KOTG	HFD,KOTG	0.2974250	0.3288969	-0.70521	1.300062	0.9423
HFD,KO	LFD,KOTG	0.2069187	0.3449500	-0.84466	1.258493	0.9902
HFD,WT	LFD,WT	0.0520200	0.3288969	-0.95062	1.054657	1.0000
HFD,WT	LFD,KO	0.0511250	0.3288969	-0.95151	1.053762	1.0000
LFD,KO	LFD,WT	0.0008950	0.3288969	-1.00174	1.003532	1.0000

Response: *Fbp2/28SrRNA*

Source	LogWorth	PValue	
Genotype	1.033	0.09278	
Diet*Genotype	0.493	0.32132	
Diet	0.465	0.34261	^

Summary of Fit

RSquare	0.235302
RSquare Adj	0.103458
Root Mean Square Error	0.927053
Mean of Response	1.066267
Observations (or Sum Wgts)	35

Level	- Level	Difference	Std Err Dif	Lower CL	Upper CL	p-Value
LFD,KO	LFD,WT	1.398007	0.5352346	-0.23365	3.029660	0.1265
LFD,KO	HFD,KOTG	1.340893	0.5352346	-0.29076	2.972547	0.1557
LFD,KO	HFD,WT	1.072658	0.5352346	-0.55900	2.704312	0.3645
LFD,KO	LFD,KOTG	0.961088	0.5352346	-0.67057	2.592742	0.4840
LFD,KO	HFD,KO	0.854767	0.5613588	-0.85653	2.566060	0.6530
HFD,KO	LFD,WT	0.543240	0.5613588	-1.16805	2.254532	0.9244
HFD,KO	HFD,KOTG	0.486126	0.5613588	-1.22517	2.197419	0.9516
LFD,KOTG	LFD,WT	0.436918	0.5352346	-1.19474	2.068572	0.9621
LFD,KOTG	HFD,KOTG	0.379805	0.5352346	-1.25185	2.011458	0.9793
HFD,WT	LFD,WT	0.325348	0.5352346	-1.30631	1.957002	0.9896
HFD,WT	HFD,KOTG	0.268235	0.5352346	-1.36342	1.899888	0.9957
HFD,KO	HFD,WT	0.217891	0.5613588	-1.49340	1.929184	0.9987
LFD,KOTG	HFD,WT	0.111570	0.5352346	-1.52008	1.743223	0.9999
HFD,KO	LFD,KOTG	0.106321	0.5613588	-1.60497	1.817614	1.0000
HFD,KOTG	LFD,WT	0.057113	0.5352346	-1.57454	1.688767	1.0000

Response: *Pink1/28SrRNA*

Source	LogWorth	PValue	
Genotype	1.609	0.02458	
Diet*Genotype	0.294	0.50871	
Diet	0.123	0.75310	^

Summary of Fit

RSquare	0.252654
RSquare Adj	0.123802
Root Mean Square Error	0.351298
Mean of Response	1.148872
Observations (or Sum Wgts)	35

Level	- Level	Difference	Std Err Dif	Lower CL	Upper CL	p-Value
LFD,KOTG	LFD,WT	0.5256433	0.2028218	-0.092655	1.143942	0.1316
HFD,KO	LFD,WT	0.4658557	0.2127213	-0.182621	1.114333	0.2728
LFD,KOTG	HFD,WT	0.4083117	0.2028218	-0.209987	1.026610	0.3596
HFD,KOTG	LFD,WT	0.3676283	0.2028218	-0.250670	0.985927	0.4738
HFD,KO	HFD,WT	0.3485240	0.2127213	-0.299953	0.997001	0.5811
LFD,KO	LFD,WT	0.3117733	0.2028218	-0.306525	0.930072	0.6441
HFD,KOTG	HFD,WT	0.2502967	0.2028218	-0.368002	0.868595	0.8167
LFD,KOTG	LFD,KO	0.2138700	0.2028218	-0.404429	0.832169	0.8951
LFD,KO	HFD,WT	0.1944417	0.2028218	-0.423857	0.812740	0.9272
LFD,KOTG	HFD,KOTG	0.1580150	0.2028218	-0.460284	0.776314	0.9689
HFD,KO	LFD,KO	0.1540823	0.2127213	-0.494395	0.802559	0.9773
HFD,WT	LFD,WT	0.1173317	0.2028218	-0.500967	0.735630	0.9917
HFD,KO	HFD,KOTG	0.0982273	0.2127213	-0.550250	0.746704	0.9971
LFD,KOTG	HFD,KO	0.0597877	0.2127213	-0.588689	0.708265	0.9997
HFD,KOTG	LFD,KO	0.0558550	0.2028218	-0.562444	0.674154	0.9998

Response: *Pgc-1α/28SrRNA*

Source	LogWorth	PValue	
Genotype	0.272	0.53484	
Diet*Genotype	0.234	0.58304	
Diet	0.210	0.61613	^

Summary of Fit

RSquare	0.087073
RSquare Adj	-0.07595
Root Mean Square Error	0.555282
Mean of Response	0.908256
Observations (or Sum Wgts)	34

Level	- Level	Difference	Std Err Dif	Lower CL	Upper CL	p-Value
LFD,KOTG	HFD,WT	0.4487800	0.3205924	-0.53091	1.428472	0.7268
HFD,KO	HFD,WT	0.4273490	0.3362402	-0.60016	1.454859	0.7978
LFD,KOTG	HFD,KOTG	0.2832250	0.3362402	-0.74428	1.310735	0.9567
HFD,KO	HFD,KOTG	0.2617940	0.3511914	-0.81140	1.334993	0.9742
LFD,KOTG	LFD,WT	0.2560500	0.3205924	-0.72364	1.235742	0.9654
LFD,KO	HFD,WT	0.2421467	0.3205924	-0.73755	1.221839	0.9727
HFD,KO	LFD,WT	0.2346190	0.3362402	-0.79289	1.262129	0.9807
LFD,KOTG	LFD,KO	0.2066333	0.3205924	-0.77306	1.186325	0.9864
LFD,WT	HFD,WT	0.1927300	0.3205924	-0.78696	1.172422	0.9901
HFD,KO	LFD,KO	0.1852023	0.3362402	-0.84231	1.212712	0.9934
HFD,KOTG	HFD,WT	0.1655550	0.3362402	-0.86195	1.193065	0.9961
LFD,KO	HFD,KOTG	0.0765917	0.3362402	-0.95092	1.104101	0.9999
LFD,KO	LFD,WT	0.0494167	0.3205924	-0.93028	1.029109	1.0000
LFD,WT	HFD,KOTG	0.0271750	0.3362402	-1.00033	1.054685	1.0000
LFD,KOTG	HFD,KO	0.0214310	0.3362402	-1.00608	1.048941	1.0000
