# Supplementary material for: Mitochondria-Targeted DNA Repair Glycosylase hOGG1 Protects Against HFD-Induced Liver Oxidative Mitochondrial DNA Damage and Insulin Resistance in OGG1-Deficient Mice
Source: Int J Mol Sci. 2024 Nov 13;25(22):12168. doi: 10.3390/ijms252212168 (PMC11595121; doi:10.3390/ijms252212168)
Supplement: Supplementary file 1 [file ijms-25-12168-s001.zip › ijms-3233700-Proof_Suppl_data.pdf]

Supplemental Information.

Supplemental Table 1. Primers for mtDNA and nDNA.

Gene	Forward	Reverse
D-Loop	5’-AATGCTACTCAATACCAA-3’	5’-TATGTCTTTCAAGTTCTTAG-3’
Nd1	5’-CCAGAACTCTACTCAACTAACTT-3’	5’- GGATAGGATGCTCGGATTC-3’
28SrRNA	5’-TCGGCTCTTCCTATCATT-3’	5-GCAACAACACATCATCAG-3’

Suplemental Table 2. Primers for quantitative reverse transcription PCR.

Gene	Forward	Reverse
Fbp2	5’- CCATCGGAACTATATTTGCTAT-3’	5’-ATCTACTCCTTGTCCTGTG-3’
Glut2	5’-TGTTCCTAACCGGGATGATT-3’	5’-GAAGATGGCAGTCATGCTCA-3’
Pdk4	5’-GAAGAAATGGTGGTAAACT-3’	5’-CATACTACTCTCACGAAAG-3’
Pgc-1α	5’-GATGACAGTGAAGATGAA-3’	5’-TAAAGGAAGAGCAAGAAG-3’
Pink1	5’-CGTCCAGTTAGGTTCTTG-3’	5’-TCAGTGATAGGTTAGTCATTAC-3’
28SrRNA	5’-TCGGCTCTTCCTATCATT-3’	5’-GCAACAACACATCATCAG-3’

Supplemental Fig.1

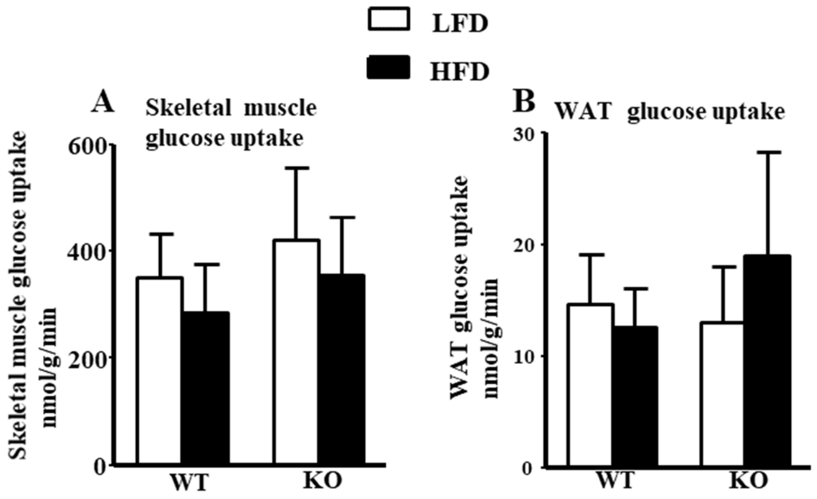

Figure 1. Tissue-specific glucose uptake was not diminished in *Ogg1-KO* mice fed HFD when compared to WT mice fed with HFD. (**A**) Insulin-stimulated glucose uptake in skeletal muscle and (**B**) epidydimal WAT. Values are the mean ± SD, n=4-7 per group, *p<0.05. Statistical analysis can be found at Supplemental File 1.

Supplemental Fig.2

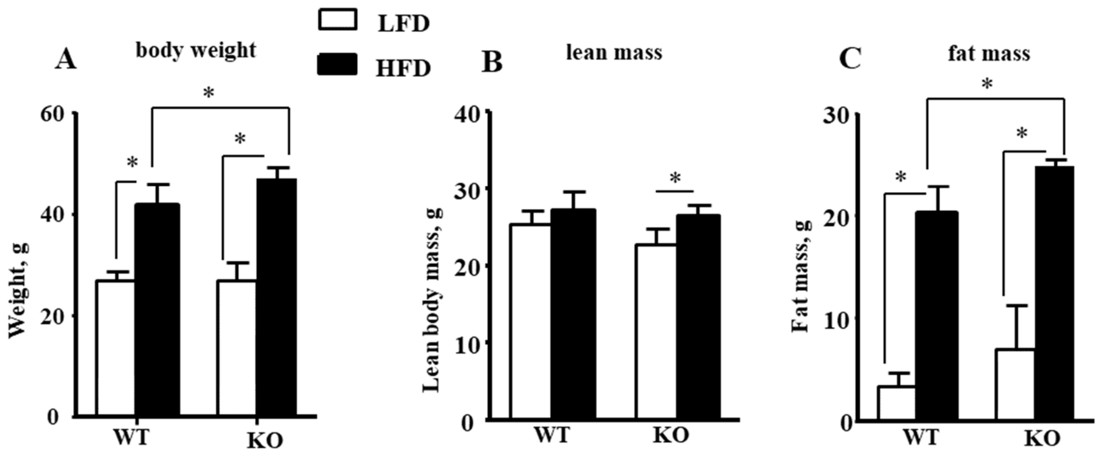

Figure 2. **Ogg1-KO mice fed HFD displayed greater obese phenotype as compared to WT mice fed HFD.** (**A**) HFD induced more weight gain in *Ogg1-KO* mice compared to WT mice. (**B**) Whole body lean mass in WT and *Ogg1-KO* mice. (**C)** Whole body fat mass was significantly increased in *Ogg1-KO* mice fed HFD compared to WT mice fed HFD. Values are the mean ± SD, n=5-8 per group, *p<0.05. Statistical analysis can be found at Supplemental File 1.

Supplemental Fig.3

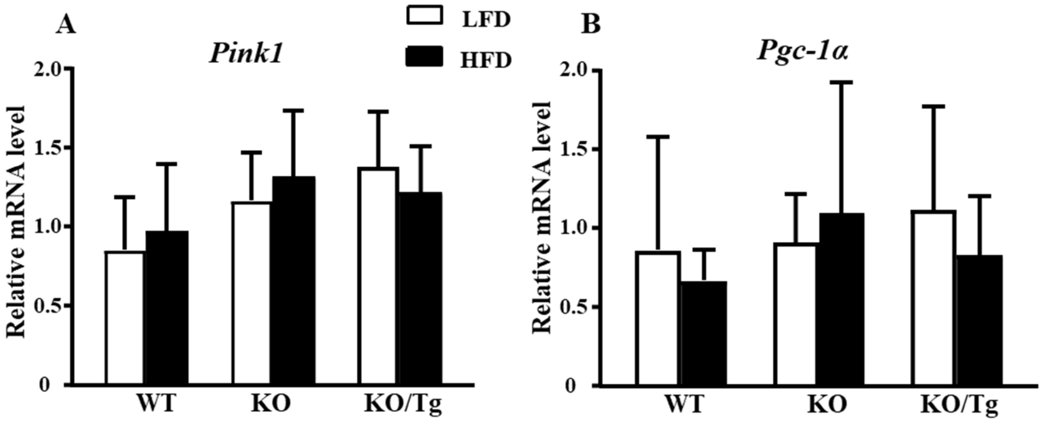

Figure 3. mRNA level of (A) *Pink1* and (B) *Pgc-1α* in WT, *Ogg1-KO* and *Ogg1-KO/Tg* mice fed either LFD or HFD. Values are the mean ± SD, n=5-6 per group. Statistical analysis can be found at Supplemental File 2.

Supplemental Fig.4

OXPHOS

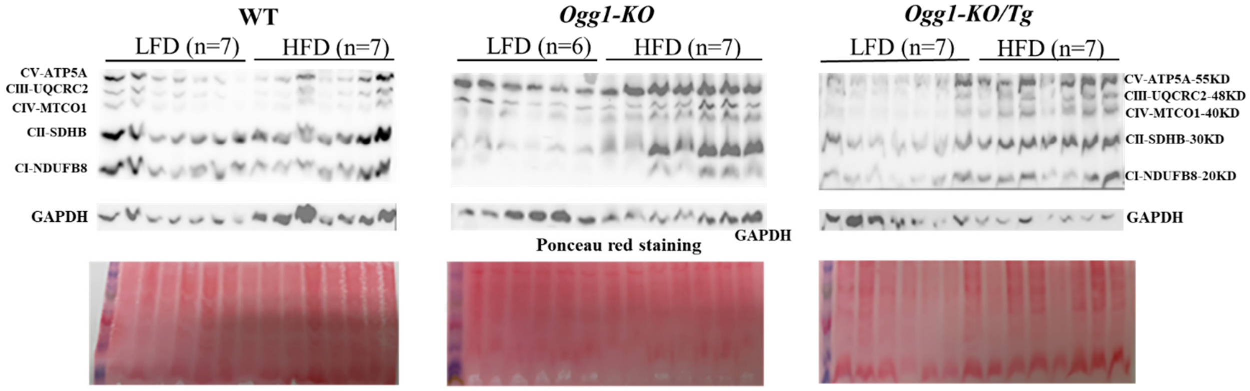

Figure 4. Expression of mitochondrial OXPHOS proteins in WT, *Ogg1-KO* and *Ogg1-KO/Tg* mice fed either LFD or HFD for 16 weeks. GAPDH was used as loading control. Membranes were stained with Ponceau S prior to blocking and incubating in primary antibody (below each immunoblots part) as an additional loading control (n=6-7).

Supplemental Fig.5

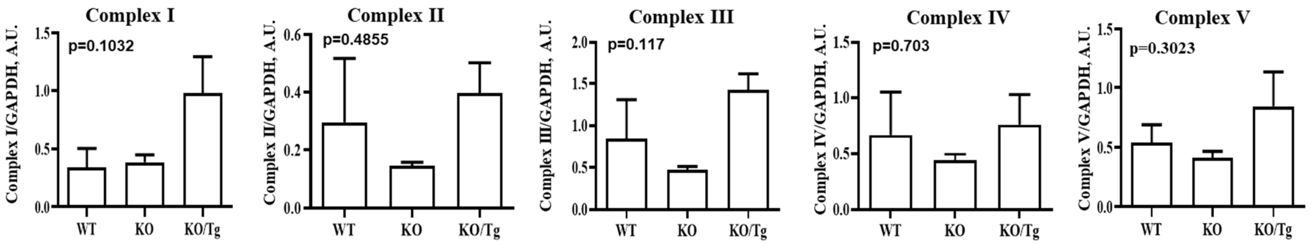

Figure 5. Densitometry analysis of Western blot for OXPHOS proteins. For densitometry, bands images were quantified and band intensities for each of five mitochondrial OXPHOS proteins were normalized to GAPDH and presented as arbitrary units (A.U.). Values are mean ± SE, n=4, p level for each complex's analysis is indicated in the corresponding graph.

Supplemental Fig.6

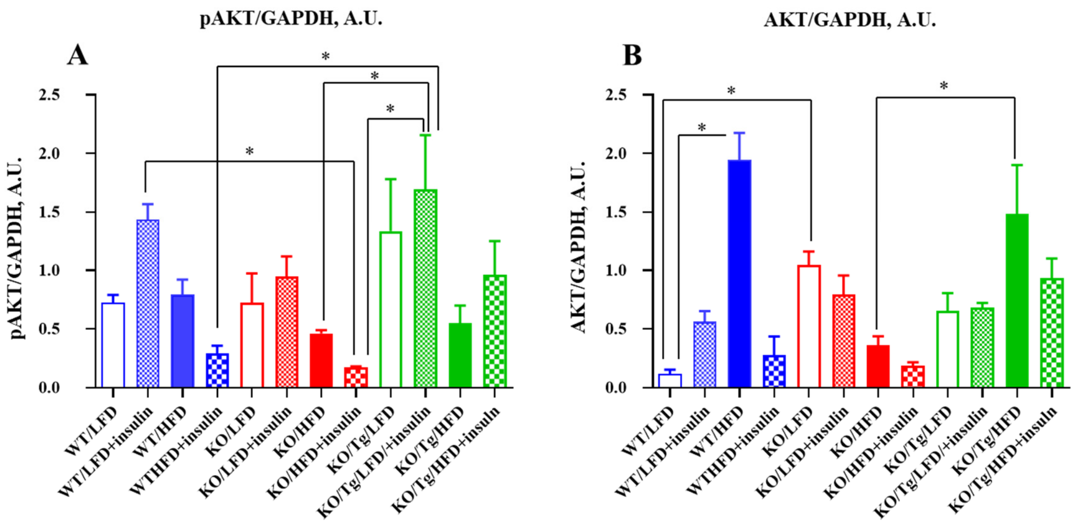

Figure 6. **Densitometry analysis of Western blots for insulin signaling data**. For densitometry analysis, bands images were quantified and band intensities for (**A**) pAKT or (**B**) AKT were normalized to GAPDH and presented as arbitrary units (A.U.) in the graphs. Values are mean ± SE, n=3, *p<0.05. Part of the statistical analysis for AKT/GAPDH densitometry with only the groups, which showed statistical difference with p<0.05 is presented in the Supplemental Table 3 below.

Supplemental Table 3. Statistical analysis for AKT/GAPDH densitometry with the groups, which showed statistical difference with p<0.05. Yes/No indicates +/- insulin, correspondingly.

Level	- Level	Difference	Std Err Dif	Lower CL	Upper CL	p-Value
WT,HFD,No	WT,LFD,No	1.828000	0.2460391	0.940874	2.715126	<.0001*
WT,HFD,No	KO,HFD,Yes	1.757000	0.2460391	0.869874	2.644126	<.0001*
WT,HFD,No	WT,HFD,Yes	1.666000	0.2460391	0.778874	2.553126	<.0001*
WT,HFD,No	KO,HFD,No	1.580667	0.2460391	0.693540	2.467793	<.0001*
WT,HFD,No	WT,LFD,Yes	1.384333	0.2460391	0.497207	2.271460	0.0004*
KOTG,HFD,No	WT,LFD,No	1.366667	0.2460391	0.479540	2.253793	0.0005*
KOTG,HFD,No	KO,HFD,Yes	1.295667	0.2460391	0.408540	2.182793	0.0010*
WT,HFD,No	KOTG,LFD,No	1.290000	0.2460391	0.402874	2.177126	0.0011*
WT,HFD,No	KOTG,LFD,Yes	1.264333	0.2460391	0.377207	2.151460	0.0014*
KOTG,HFD,No	WT,HFD,Yes	1.204667	0.2460391	0.317540	2.091793	0.0025*
WT,HFD,No	KO,LFD,Yes	1.150667	0.2460391	0.263540	2.037793	0.0043*
KOTG,HFD,No	KO,HFD,No	1.119333	0.2460391	0.232207	2.006460	0.0058*
WT,HFD,No	KOTG,HFD,Yes	1.009000	0.2460391	0.121874	1.896126	0.0165*
KO,LFD,No	WT,LFD,No	0.927000	0.2460391	0.039874	1.814126	0.0351*
KOTG,HFD,No	WT,LFD,Yes	0.923000	0.2460391	0.035874	1.810126	0.0364*
WT,HFD,No	KO,LFD,No	0.901000	0.2460391	0.013874	1.788126	0.0443*

Supplemental Fig.7

Methylation of total DNA (5-mC)

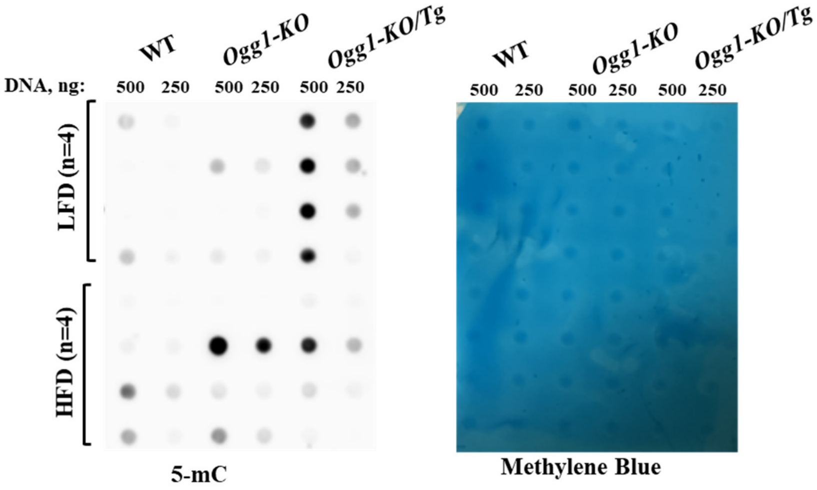

Figure 7. Methylation DNA (5-mC) in WT, *Ogg1-KO* and *Ogg1-KO/Tg* mice fed either LFD or HFD. Representative dot blot for 5-mC using total DNA isolated from liver (n=4). The methylene blue staining of total genomic DNA was used as a loading control.
